# Activated cGAS/STING signaling elicits endothelial cell senescence in early diabetic retinopathy

**DOI:** 10.1172/jci.insight.168945

**Published:** 2023-06-22

**Authors:** Haitao Liu, Sayan Ghosh, Tanuja Vaidya, Sridhar Bammidi, Chao Huang, Peng Shang, Archana Padmanabhan Nair, Olivia Chowdhury, Nadezda A. Stepicheva, Anastasia Strizhakova, Stacey Hose, Nikolaos Mitrousis, Santosh Gopikrishna Gadde, Thirumalesh MB, Pamela Strassburger, Gabriella Widmer, Eleonora M. Lad, Patrice E. Fort, José-Alain Sahel, J. Samuel Zigler, Swaminathan Sethu, Peter D. Westenskow, Alan D. Proia, Akrit Sodhi, Arkasubhra Ghosh, Derrick Feenstra, Debasish Sinha

**Affiliations:** 1 Department of Ophthalmology, University of Pittsburgh School of Medicine, Pittsburgh, Pennsylvania, USA.; 2 GROW Laboratory, Narayana Nethralaya Foundation, Bengaluru, India.; 3 Pharma Research and Early Development, Roche Innovation Center Basel, F. Hoffmann-La Roche, Ltd., Basel, Switzerland.; 4 Doheny Eye Institute, Los Angeles, California, USA.; 5 Department of Ophthalmology, Duke University Medical Center, Durham, North Carolina, USA.; 6 Kellogg Eye Center, University of Michigan School of Medicine, Ann Arbor, Michigan, USA.; 7 Institut De La Vision, INSERM, CNRS, Sorbonne Université, Paris, France.; 8 Wilmer Eye Institute, The Johns Hopkins University School of Medicine, Baltimore, Maryland, USA.; 9 Department of Pathology, Duke University Medical Center, Durham, North Carolina, USA.; 10 Department of Pathology, Campbell University Jerry M. Wallace School of Osteopathic Medicine, Lillington, North Carolina, USA.

**Keywords:** Ophthalmology, Cellular senescence, Retinopathy, Signal transduction

## Abstract

Diabetic retinopathy (DR) is a leading cause of blindness in working-age adults and remains an important public health issue worldwide. Here we demonstrate that the expression of stimulator of interferon genes (STING) is increased in patients with DR and animal models of diabetic eye disease. STING has been previously shown to regulate cell senescence and inflammation, key contributors to the development and progression of DR. To investigate the mechanism whereby STING contributes to the pathogenesis of DR, diabetes was induced in *STING*-KO mice and *STING*^GT^ (loss-of-function mutation) mice, and molecular alterations and pathological changes in the retina were characterized. We report that retinal endothelial cell senescence, inflammation, and capillary degeneration were all inhibited in *STING*-KO diabetic mice; these observations were independently corroborated in *STING*^GT^ mice. These protective effects resulted from the reduction in TBK1, IRF3, and NF-κB phosphorylation in the absence of STING. Collectively, our results suggest that targeting STING may be an effective therapy for the early prevention and treatment of DR.

## Introduction

Diabetic retinopathy (DR) is the most common microvascular complication of diabetes mellitus and remains one of the main causes of visual impairment and blindness in middle-aged and elderly individuals (1). Traditionally regarded as microvascular disease, DR is characterized by capillary degeneration, pericyte loss, and vascular leakage (2–5). DR can progress from a mild, nonproliferative stage to moderate and then severe stages. Sufficient damage to retinal vessels can result in nonperfusion, ischemia, and the expression of angiogenic mediators (e.g., vascular endothelial growth factor or VEGF). These angiogenic mediators, in turn, promote the formation of fragile retinal blood vessels (i.e., retinal neovascularization), heralding the development of proliferative DR (PDR). If untreated, PDR results in bleeding, scarring, and profound vision loss (6). Current therapies available for DR, including anti-VEGF agents and invasive laser treatments, are helpful for patients with advanced stages of DR. However, this leaves most of the diabetic population who suffer from earlier stages of DR without any treatment options (7). Consequently, DR remains a tremendous vision-threatening burden on the growing diabetic population, highlighting the importance of identifying early steps in the development of diabetic eye disease.

In this regard, emerging evidence suggests that cellular senescence (cessation of cell division) in the retina may contribute to the development of DR (8). Endothelial cells cultured in high glucose exhibit increased β-galactosidase activity, a marker of cellular senescence (8). Premature senescence of retinal endothelial cells (RECs) was also demonstrated in streptozotocin-induced (STZ-induced) hyperglycemic mice, reflected by increased mRNA levels of senescence markers, including p16^INK4a^, p21, and p53. Likewise, accumulation of senescent cells has been documented in the retinas of patients with DR (9). While these elegant studies provide a strong link between cellular senescence and DR, the molecular mechanism(s) by which cellular senescence contributes to the development of DR remain unknown.

There is also increasing evidence that inflammatory responses play a crucial role in the development of DR (10–12). Many retinal glial and neuronal cells, including Müller cells, astrocytes, microglia, photoreceptor cells, and ganglion cells have been reported to contribute to the elevated levels of retinal inflammatory molecules VEGF, monocyte chemoattractant protein-1 (MCP-1), inducible nitric oxide synthase (iNOS), cyclooxygenase-2 (COX-2), IL-1β, and NF-κB in diabetic patients and animals (12–17). Conversely, antiinflammatory therapies (e.g., high-dose aspirin) and germline deletion of iNOS have been shown to inhibit retinal capillary degeneration in diabetic animals, further strengthening the link between inflammation and DR pathogenesis (18–20). In addition, the retinal pigment epithelium and inflammatory cells (e.g., neutrophils) have also been implicated in DR pathogenesis by elevating intercellular adhesion molecule 1 (ICAM-1) and neutrophil elastase levels, which leads to retinal vascular damage in diabetic mice (4, 21, 22). While DR exhibits features of chronic neuroinflammation (23) and inflammatory cytokines have been shown to activate cellular senescence (24–26), senescent cells also contribute to inflammation and ICAM-1 expression in diabetes (27, 28). Collectively, these observations demonstrate the important relationship between inflammatory alterations and endothelial cellular senescence for DR pathogenesis. Identifying the signaling pathway(s) that link cellular senescence and inflammation in DR may therefore expose novel targets for its treatment at the early stages of the disease.

Of interest, the cyclic GMP–AMP synthase (cGAS)/stimulator of interferon (IFN) genes (STING) signaling pathway is a critical part of the innate immune response to viral and bacterial infections (29). This pathway is activated when cytosolic DNA from pathogenic microorganisms or damaged cells is detected by the enzyme cGAS, which catalyzes the synthesis of cyclic GMP-AMP (cGAMP). cGAMP then binds to and activates the STING protein, initiating a downstream signaling cascade that ultimately leads to the production of type I IFNs and other proinflammatory cytokines (30). Dysregulation of the cGAS/STING pathway has been implicated in a variety of diseases, including diabetes, lupus, arthritis, and some types of cancer (29–32).

The cGAS/STING signaling pathway has been shown to regulate both cellular senescence and inflammation, thus presenting itself as a possible link between these processes during the pathogenesis of DR (33). There are multiple factors associated with DR that can activate the cGAS/STING signaling pathway, including extracellular self-DNA, cell-intrinsic genomic DNA, and mitochondrial DNA (34–36). Activation of STING leads to cellular senescence by activating the kinase tank-binding kinase 1 (TBK1), which in turn phosphorylates the transcription factor IFN regulatory factor 3 (IRF3) and contributes to the production of type I IFNs (37–39). Type I IFNs stimulate cell senescence in endothelial cells, a key event in the pathogenesis of DR (40). Collectively, these observations suggest that the accumulation of extracellular and cytoplasmic DNA in DR may cause cGAS/STING activation and lead to REC senescence through secretion of type I IFNs.

The cGAS/STING signaling pathway also regulates inflammation, a known inducer of cell senescence (29). Activation of STING stimulates the kinase IκB kinase complex (IKK), leading to the phosphorylation of inhibitor of NF-κB (IκB), and activation of the NF-κB signaling pathway. In turn, NF-κB stimulates the expression of inflammatory cytokines, including tumor necrosis factor (TNF), IL-1β, IL-6, and IL-8 (29), which have been previously implicated both in DR (4, 41) and the initiation and maintenance of cellular senescence (42). Based on published data, we hypothesized that blocking activation of STING could decrease retinal inflammatory cytokines, which in turn would inhibit diabetes-induced endothelial cell senescence and damage to the retinal vasculature. In the current study, we used patient tissue and animal models of diabetic eye disease to investigate the role of the cGAS/STING pathway in DR and assessed the therapeutic potential of inhibiting this signaling pathway in early stages of the disease to delay the progression of DR. We demonstrate the importance of the STING signaling pathway in regulating endothelial cellular senescence, inflammation, and oxidative stress in DR pathogenesis. Furthermore, we provide a strategy for targeting the cGAS/STING pathway as a future therapy for DR.

## Results

### STING expression is increased in the retina of DR donor eyes.

**Figure 1:**
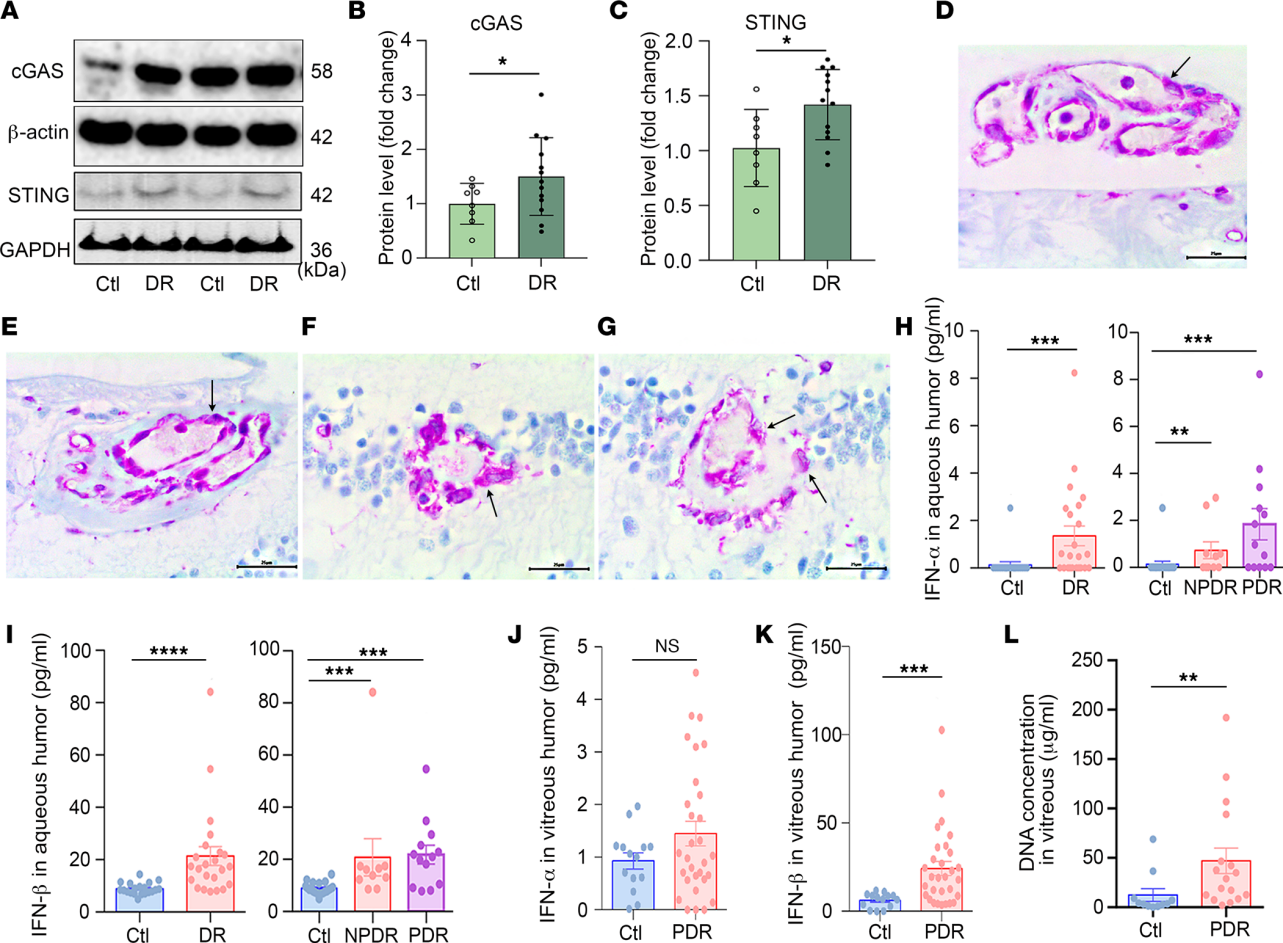
Elevated cGAS, STING, type I IFN, and free DNA levels in patients with diabetic retinopathy. Representative immunoblots (**A**) and densitometry graphs show that the protein levels of cGAS (**B**) and STING (**C**) are increased in the retina of human DR donor cadaver tissue (Ctl, *n* = 8 DR, *n* = 13). Immunostaining of STING in human PDR donor retinal sections shows that (**D**) STING is present (arrow) in endothelial cells, (**E**) intraretinal neovascularization, (**F**) faintly in an occluded capillary and strongly in surrounding microglia/macrophages, and (**G**) in endothelial cells and surrounding microglia/macrophages of a hyalinized capillary microaneurysm. Scale bars: 25 μm. The levels of IFN-α (**H**) and IFN-β (**I**) in the aqueous humor, and IFN-α (**J**) and IFN-β (**K**) in the vitreous humor of patients without (Ctl) and with DR or PDR. (Ctl, *n* = 20, DR, *n* = 23; NPDR, *n* = 10; PDR, *n* = 13 for **H** and **I**; Ctl, *n* = 13, PDR, *n* = 30 for **J** and **K**). (**L**) The levels of free DNA in the vitreous humor of patients without (*n* = 11) and with PDR (*n* = 17). Data represent mean ± SEM. *P < 0.05; **P < 0.01; ***P < 0.001; ****P < 0.0001 by Mann-Whitney *U* test for **B**, **C**, **H** (left), **I** (left), and **J**–**L**, or Kruskal-Wallis test for **H** (right) and **I** (right). Ctl, control; NPDR, nonproliferative diabetic retinopathy; PDR, proliferative diabetic retinopathy.

To evaluate the role of the cGAS/STING pathway in DR, we first examined STING and cGAS levels in human retina. To this end, we performed Western blot analysis on human retina cadaver tissue lysates from patients with DR and observed increased expression of STING and cGAS in the retina of donors with DR compared with nondiabetic control donors (Figure 1, A–C, and Supplemental Table 1; supplemental material available online with this article; https://doi.org/10.1172/jci.insight.168945DS1). Furthermore, we performed immunostaining for STING protein (purple stain) and found it localized to endothelial cells, including those found in preretinal neovascular membranes (Figure 1D), intraretinal neovascularization (Figure 1E), faintly in an occluded capillary, and strongly in surrounding microglia/macrophages (Figure 1F) in endothelial cells. Expression was also observed surrounding microglia/macrophages of a hyalinized capillary microaneurysm (Figure 1G; hyalinized vessel wall separates the endothelial cells from the surrounding microglia/macrophages).

STING has previously been reported to regulate the expression of type I IFNs (α and β) (43). To examine whether the increased expression of STING observed in donor eyes with DR correlated with an increase in expression of type I IFNs, we obtained aqueous humor samples from patients with DR and performed ELISAs for IFN-α and IFN-β. We observed significantly elevated levels of both IFN-α and IFN-β in the aqueous humor of DR patients when compared with nondiabetic controls (Figure 1, H and I, and Supplemental Table 2). These results were corroborated for IFN-β (but not IFN-α) in the vitreous humor of a separate cohort of patients with PDR who underwent vitrectomy surgery, compared with nondiabetic controls (Figure 1, J and K, and Supplemental Table 3). The increase in IFNs correlated with an increase in DR-associated angiogenic and inflammatory factors (e.g., VEGF, MCP-1, IL-8, and IFN-γ) in both the aqueous (Supplemental Figure 1, A–D) and vitreous humors (Figure 1, E–G). These vasoactive and/or inflammatory mediators have previously been implicated in the promotion of retinopathy of patients with diabetic eye disease. Next, we performed immunohistochemical staining for IFN-β protein in postmortem eyes from patients without diabetes, those with diabetes but no ocular histological changes suggestive of retinopathy, and eyes from patients with diabetes and nonproliferative DR (NPDR) or PDR. All groups had IFN-β expression in cells of the iris anterior epithelial layer/dilator pupillae muscle (Supplemental Figure 1H), patchy staining of the iris anterior border layer, pigmented epithelium of the ciliary body pars plicata (Supplemental Figure 1I) and pars plana, peripheral retinal pigment epithelium in areas of ischemic atrophy and cystoid degeneration (Supplemental Figure 1J), and choroidal macrophages and melanocytes (Supplemental Figure 1K). Eyes from patients with NPDR or PDR had IFN-β staining with iris stromal clump cells in 14 of 16 eyes (Supplemental Figure 1L). Macrophages and/or melanophages (macrophages containing engulfed melanin granules) expressing IFN-β were present in 4 eyes from patients with NPDR and 3 with PDR that were treated with pan-retinal laser photocoagulation (Supplemental Figure 1M). The presence of IFN-β in intraocular tissues containing melanin may reflect melanin binding of soluble IFN-β (44, 45), which is synthesized by these layers (46). While only iris clump cell and intraretinal macrophage/melanophage IFN-β expression distinguished eyes from patients with NPDR or PDR compared with controls, this observation was adequate to help explain the increased aqueous and vitreous humor levels of IFN-β in DR eyes.

Previous studies have demonstrated that the cGAS/STING pathway can be induced by free-floating extracellular DNA (33, 47). To determine whether extracellular DNA could be the stimulus for STING expression in patients with DR, we measured free-floating DNA in the vitreous humor of diabetic patients and observed significantly higher levels of extracellular DNA in patients with PDR compared with nondiabetic controls (Figure 1L). Taken together, these data support a role for increased free-floating extracellular DNA in diabetic eyes in the activation of the cGAS/STING pathway and, in turn, IFNs.

### cGAS/STING is activated in the retinas of diabetic mice.

**Figure 2:**
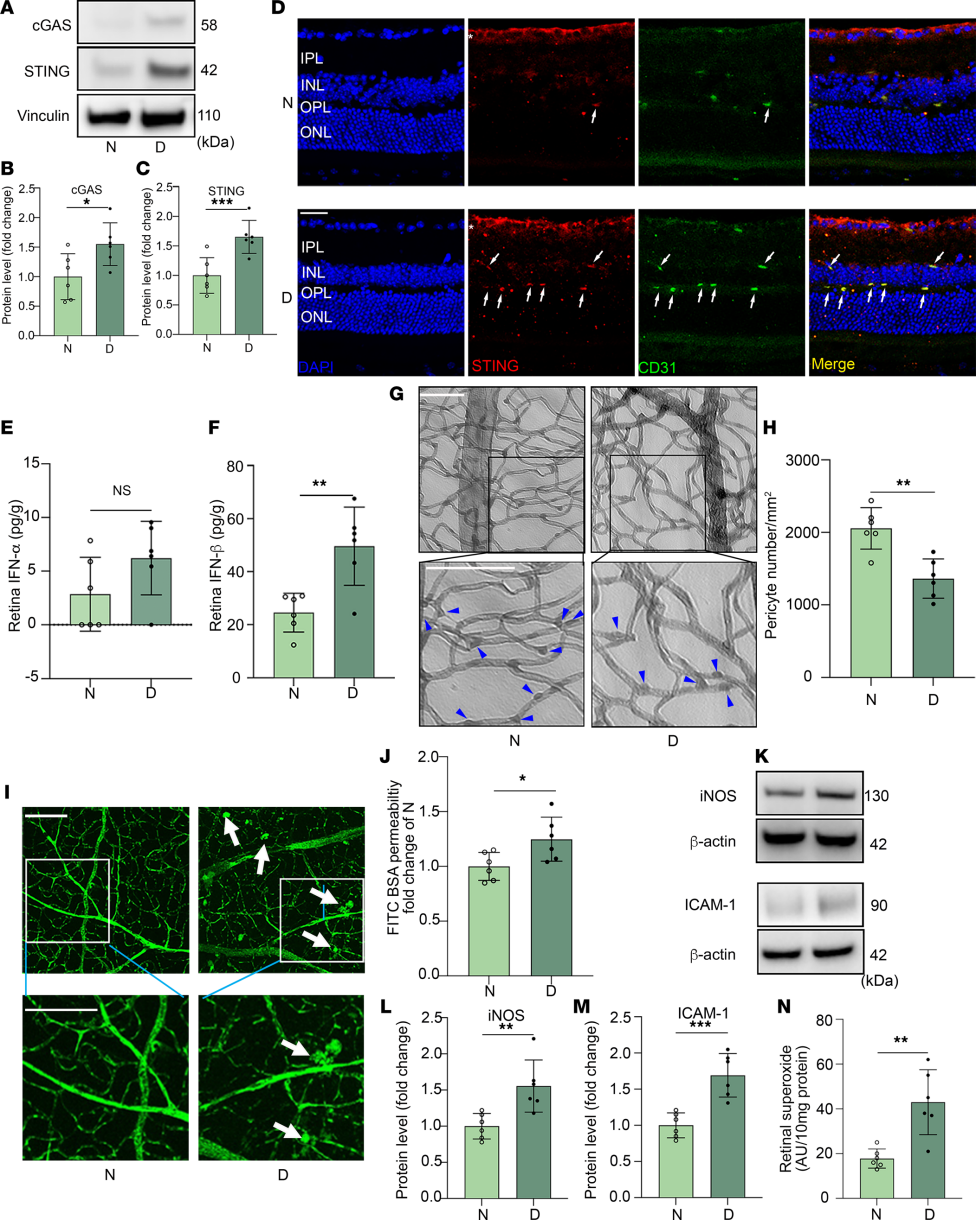
cGAS/STING signaling pathway is activated in the retina of diabetic mice. Representative immunoblots (**A**) and densitometry graphs show that the protein levels of (**B**) cGAS and (**C**) STING expression was increased in the retina of diabetic (D) mice (diabetes induction for 2 months) compared with nondiabetic (N) controls. Immunofluorescence study shows that (**D**) STING is localized in the retinal nerve fiber layer (asterisk) of both nondiabetic and diabetic mice, and more were observed in retinal endothelial cells in diabetic retina. Red, STING^+^ cells; green, CD31^+^ endothelial cells. Scale bar: 50 μm. ELISA shows that (**E**) IFN-α (not significant) and (**F**) IFN-β (significant) were increased in the diabetic retina compared with nondiabetic controls, which is correlated with pericytes loss (**G** and **H**; arrowheads indicate pericytes). (**I**) Flat-mount micrographs from WT diabetic mice (2 months of diabetes, 4 months of age) and age-matched nondiabetic (N) controls showing blood vessel hyperpermeability; arrows indicate leakage sites. Scale bars: 50 μm. (**J**) Quantification of FITC-BSA leakage into the retina. Representative immunoblots (**K**) show increased inflammatory factors iNOS (**L**) and ICAM-1 (**M**) and superoxide production (**N**) in diabetic mice. In **A**–**N**, *n* = 6 for each group. Data are expressed as mean ± SD. *P < 0.05, **P < 0.01, ***P < 0.001 versus nondiabetic controls by unpaired, 2-tailed Student’s *t* test.

The compelling human data demonstrating increased expression of STING and STING-regulated genes in patients with DR prompted us to explore whether the STING pathway could contribute to the development or progression of DR. To evaluate the contribution of STING to DR pathogenesis, we induced diabetes in 2-month-old wild-type (WT) C57BL/6J mice by i.p. injections of STZ for 5 consecutive days at a concentration of 60 mg/kg (body weight). After 2 months of sustained hyperglycemia (serum glucose > 275 mg/dL), we found increased expression of both cGAS and STING in the retina of diabetic mice compared with nondiabetic mice (Figure 2, A–C). Immunofluorescent staining showed positive STING expression in the retinal nerve fiber layer of both diabetic and nondiabetic animals, with prominent changes in the retinal blood vessels in diabetic animals compared with nondiabetics (Figure 2D), consistent with what we observed in human eyes. In addition, we found that the level of IFN-β (but not IFN-α) was increased in the retina of diabetic mice when compared with nondiabetic mice (Figure 2, E and F), similar to what was observed in vitreous samples from patients with DR. Importantly, the elevated protein level of STING correlates with pericyte loss, endothelial cell hyperpermeability, and increases in DR-associated inflammatory factors iNOS, ICAM-1, and superoxide in diabetic mice (Figure 2, G–N). These data confirm that the cGAS/STING signaling pathway is activated in the retina of diabetic mice and support a role for this pathway in the pathogenesis of DR.

### Genetic deletion of STING attenuates diabetes-induced retinal capillary degeneration and vascular leakage.

**Figure 3:**
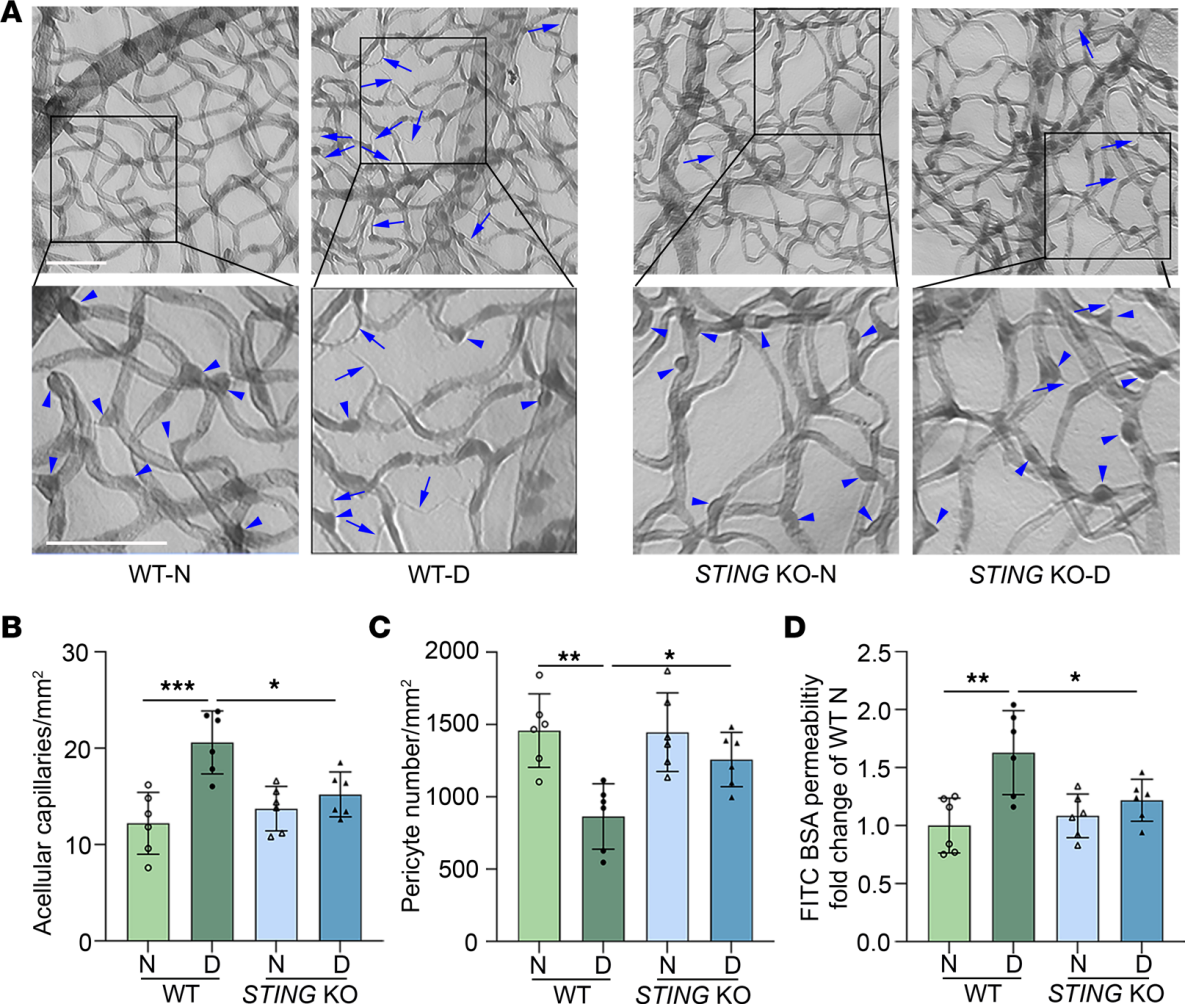
*STING* KO attenuates diabetes-induced retinal capillary degeneration and vascular leakage. (**A**) Representative micrographs of retinal vessels from diabetic mice (8 months of diabetes) and age-matched nondiabetic mice. Arrows indicate degenerated capillaries and arrowheads indicate capillary pericytes. Scale bars: 50 μm. (**B**) Diabetes increased the number of degenerated capillaries and (**C**) decreased the number of retinal capillary pericytes in WT diabetic mice compared with nondiabetic controls. *STING* KO attenuated such alterations caused by diabetes. (**D**) Diabetes-induced accumulation of FITC-BSA in the mouse retina was significantly reduced in *STING*-KO diabetic mice compared with WT diabetic controls. *n* = 6 mice for each group; the data are expressed as mean ± SD. Statistical differences were examined by ordinary 1-way ANOVA followed by Tukey’s multiple-comparison test. *P < 0.05; **P < 0.01; ***P < 0.001. N, control; KO, knockout.

To further investigate the role of STING in the pathogenesis of DR, we examined the retinal vascular changes in *STING*-KO diabetic mice. STING protein loss in KO mice was first verified by immunoblotting (Supplemental Figure 2) using leukocytes isolated from both *STING*-KO and control mice. We found an increase in acellular capillaries (Figure 3A, arrows) and a decrease in capillary pericytes (Figure 3A, arrowheads) in the retinas of WT mice after 8 months of diabetes as compared with nondiabetic controls. However, both capillary degeneration and pericyte loss were attenuated in *STING*-KO diabetic mice compared with WT (Figure 3, B and C), supporting a role for STING in retinal capillary injury in diabetic eye disease.

Injury to the retinal vasculature in diabetic mice results in vascular hyperpermeability (48, 49) that is remarkably similar to that observed in patients with diabetic eye disease (50). To evaluate whether retinal vascular leakage is affected in *STING*-KO mice, we measured the leakage of fluorescein isothiocyanate–labeled bovine serum albumin (FITC-BSA) from the retinal vasculature into the neurosensory retina, as we have previously reported (4). We found that leakage of FITC-BSA into the retina was significantly increased in WT mice after 8 months of diabetes (10 months chronic age) compared with age-matched nondiabetic controls, as previously reported (4). However, this leakage was markedly diminished in *STING*-KO diabetic mice (Figure 3D). These data suggest that STING contributes to hyperglycemia-induced retinal vascular damage and, in turn, impaired retinal vascular function in DR.

### STING loss-of-function mutation attenuates diabetes-induced retinal capillary degeneration and vascular leakage.

**Figure 4:**
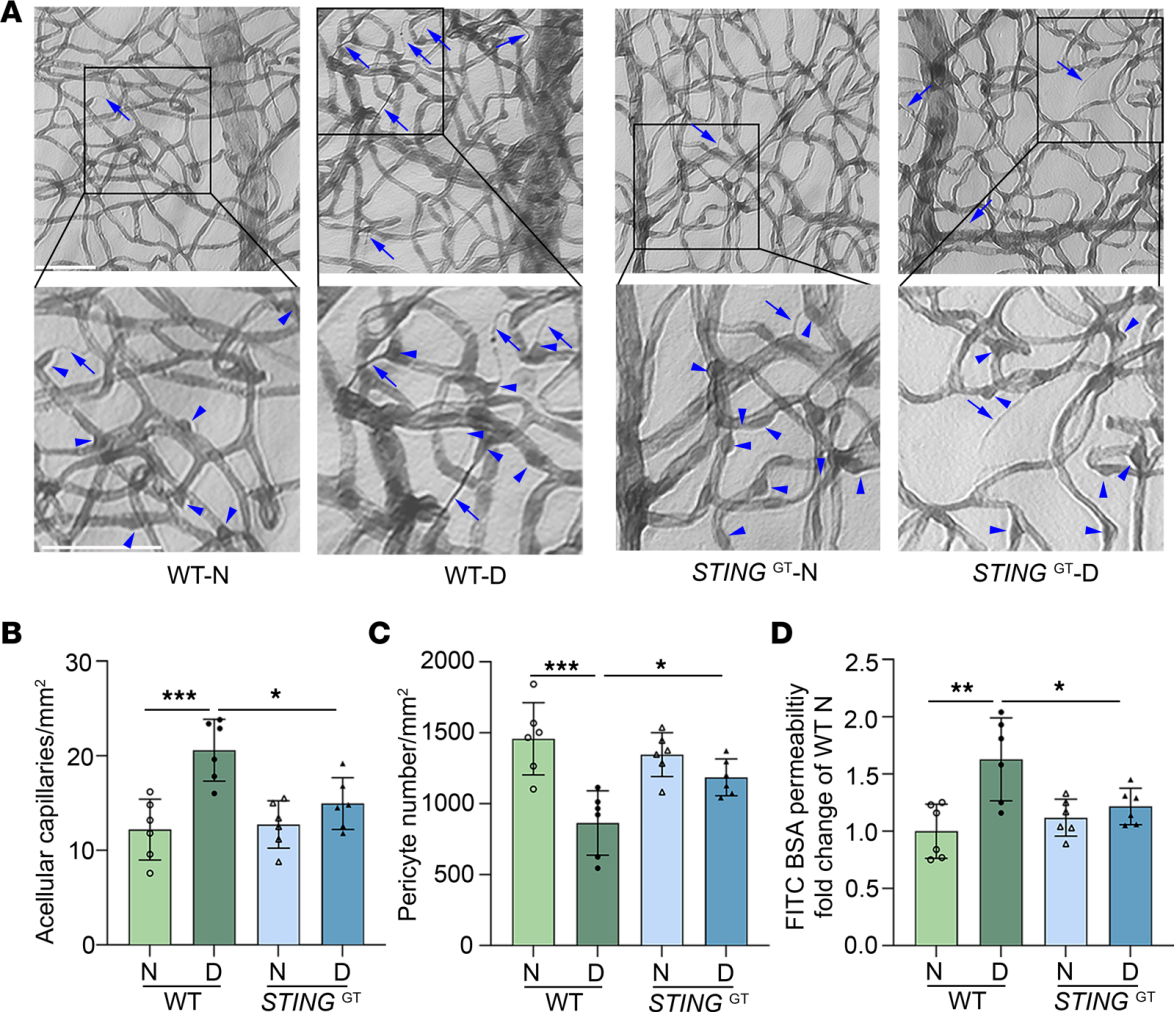
*STING*^GT^ attenuates diabetes-induced retinal capillary degeneration and vascular leakage. (**A**) Representative micrographs of retinal vessels from diabetic mice (8 months of diabetes) and age-matched nondiabetic mice. Arrows indicate degenerated capillaries and arrowheads indicate capillary pericytes. Scale bars: 50 μm. (**B**) Diabetes increased the number of degenerated capillaries and (**C**) decreased the number of retinal capillary pericytes in WT diabetic mice compared with nondiabetic controls. In *STING*^GT^ mice, such alterations caused by diabetes were attenuated. (**D**) Diabetes-induced accumulation of FITC-BSA in the mouse retina was significantly reduced in *STING*^GT^ diabetic mice compared with WT diabetic controls. *n* = 6 mice for each group; the data are expressed as mean ± SD. Statistical differences were examined by ordinary 1-way ANOVA followed by Tukey’s multiple-comparison test. *P < 0.05; **P < 0.01; ***P < 0.001. N, control; KO, knockout.

To further establish the role of STING in retinal vascular function in diabetic animals, we took advantage of the *STING* Golden Ticket mice (*STING*^GT^, loss-of-function mutation). We first verified that STING protein expression is undetectable in *STING*^GT^ mice using a leukocyte sample (Supplemental Figure 2). We then induced diabetes in *STING*^GT^ mice and examined their retinas after 8 months. Similar to *STING*-KO mice, diabetic *STING*^GT^ mice exhibited fewer acellular capillaries (Figure 4, A and B) and increased survival of capillary pericytes (Figure 4C) compared with diabetic WT mice. Accordingly, diabetes-induced retinal vascular leakage was attenuated in *STING*^GT^ diabetic mice (Figure 4D). Collectively, the data from *STING*-KO and *STING*^GT^ mice strongly support a role for STING in promoting retinal vascular damage in DR, and suggest that targeting STING may be an effective therapeutic intervention for the prevention or treatment of DR.

### Blocking STING attenuates diabetes-induced inflammatory protein synthesis, superoxide production, and leukostasis in the mouse retina.

**Figure 5:**
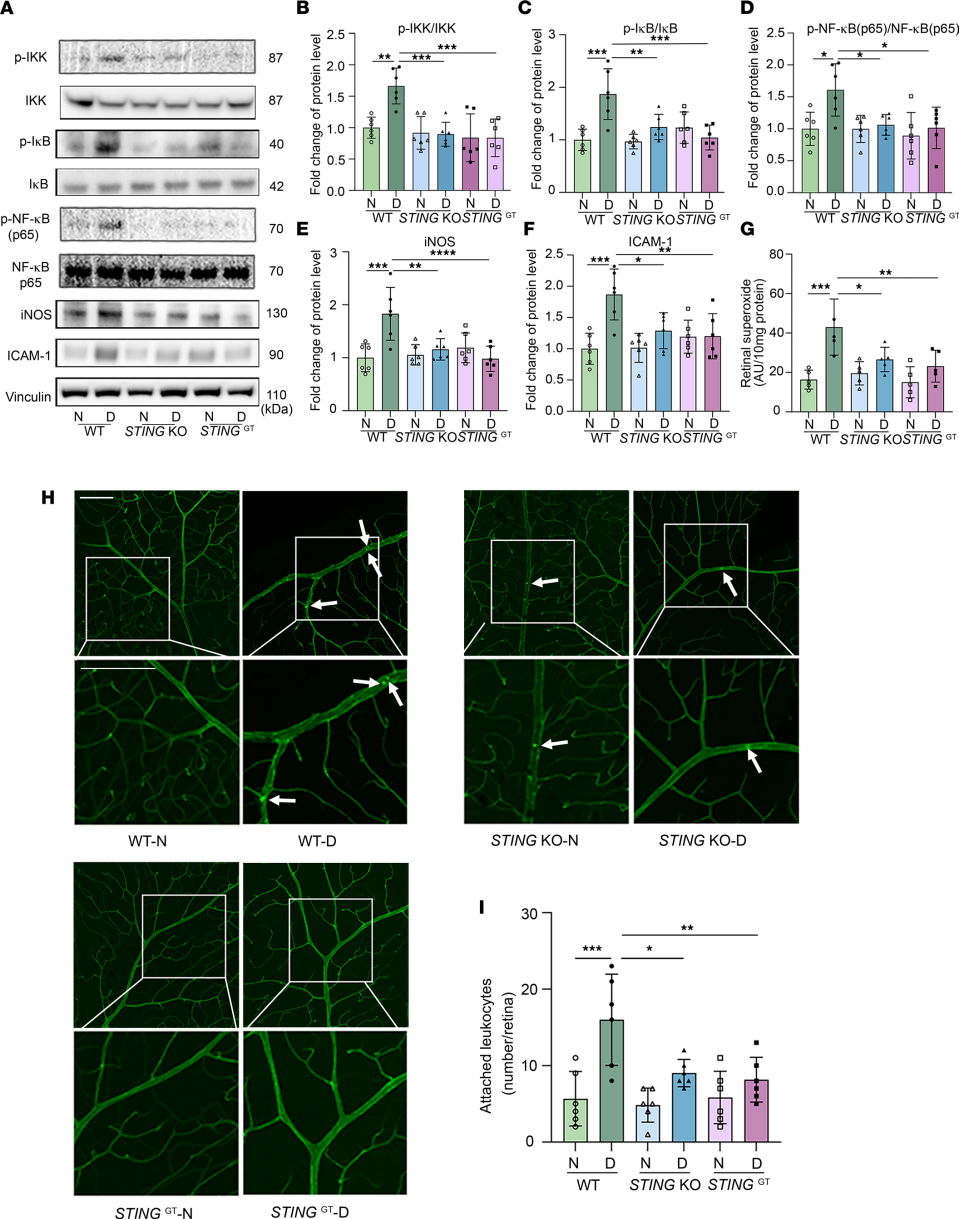
Genetic inhibition of STING inhibits NF-κB signaling and attenuates diabetes-induced increases in inflammatory proteins, production of superoxide, and leukostasis in the mouse retina. (**A**) Representative immunoblots and densitometry graphs demonstrating the diabetes-induced increases in the ratios of (**B**) p-IKK/total IKK, (**C**) p-IκB/IκB, and (**D**) p-NF-κB/total NF-κB. Levels of (**E**) iNOS and (**F**) ICAM-1 were inhibited in the retina of *STING*-KO and *STING*^GT^ diabetic mice. (**G**) Retinal superoxide was measured using lucigenin; *STING*-KO and *STING*^GT^ attenuate the retinal production of superoxide caused by diabetes. (**H**) Representative images and (**I**) quantification of attached leukocytes (arrows) in the retina blood vessels show that diabetes increased the number of adherent leukocytes in the WT diabetic retina compared with nondiabetic control mice. This number was markedly reduced in *STING*-KO and *STING*^GT^ diabetic mice. Scale bars: 100 μm. In **A**–**I**, *n* = 6 mice for each group, data are expressed as mean ± SD. Statistical differences were examined by ordinary 1-way ANOVA followed by Tukey’s multiple-comparison test. *P < 0.05, **P < 0.01, ***P < 0.001, ****P < 0.0001 versus WT nondiabetic (N) controls. KO, knockout; D, diabetes.

STING activation has been reported to trigger an NF-κB–regulated inflammatory response (43, 51). As inflammation plays a critical role in the pathogenesis of DR (11, 23, 52), we quantified phosphorylated and total levels of key components of the NF-κB signaling pathway (i.e., IKK, IκB, and NF-κB p65) as well as the expression of the NF-κB–regulated adhesion molecule ICAM-1 and inflammatory protein (iNOS, etc.) expression in diabetic and control mice. We found that in diabetic *STING*-KO and *STING*^GT^ mice, the diabetes-induced elevation of p-IκB/total IκB, p-IKK/total-IKK, and p-p65 NF-κB/total NF-κB was inhibited (Figure 5, A–D). In addition, diabetes-induced increases in retinal inflammatory proteins iNOS (Figure 5E), IL-1α, and IL-10 (Supplemental Figure 3), and ICAM-1 (Figure 5, E and F) were also inhibited in *STING*-KO and *STING*^GT^ diabetic mice. iNOS, particularly from leukocytes (53), is known to contribute to superoxide production, which is a significant contributor to retinal capillary degeneration in diabetes (53). Thus, we further investigated reactive oxygen species (ROS) in our mouse model and found genetic inhibition of STING inhibited ROS production caused by diabetes (Figure 5G).

Previous research has demonstrated that leukocytes play a significantly role in retinal vascular lesions in DR (21, 22). Studies have found that deleting *Elane* in neutrophils can attenuate diabetes-induced retinal leukostasis and vascular damage in mice (21, 22). Interestingly, the number of bound leukocytes in the retinal blood vessels from *STING*-KO and *STING*^GT^ diabetic mice was significantly lower than in WT diabetic mice (Figure 5, H and I), supporting a role for STING in retinal leukostasis in diabetic eye disease.

### Increased expression of STING causes cellular senescence in endothelial cells through increased secretion of IFN-β.

**Figure 6:**
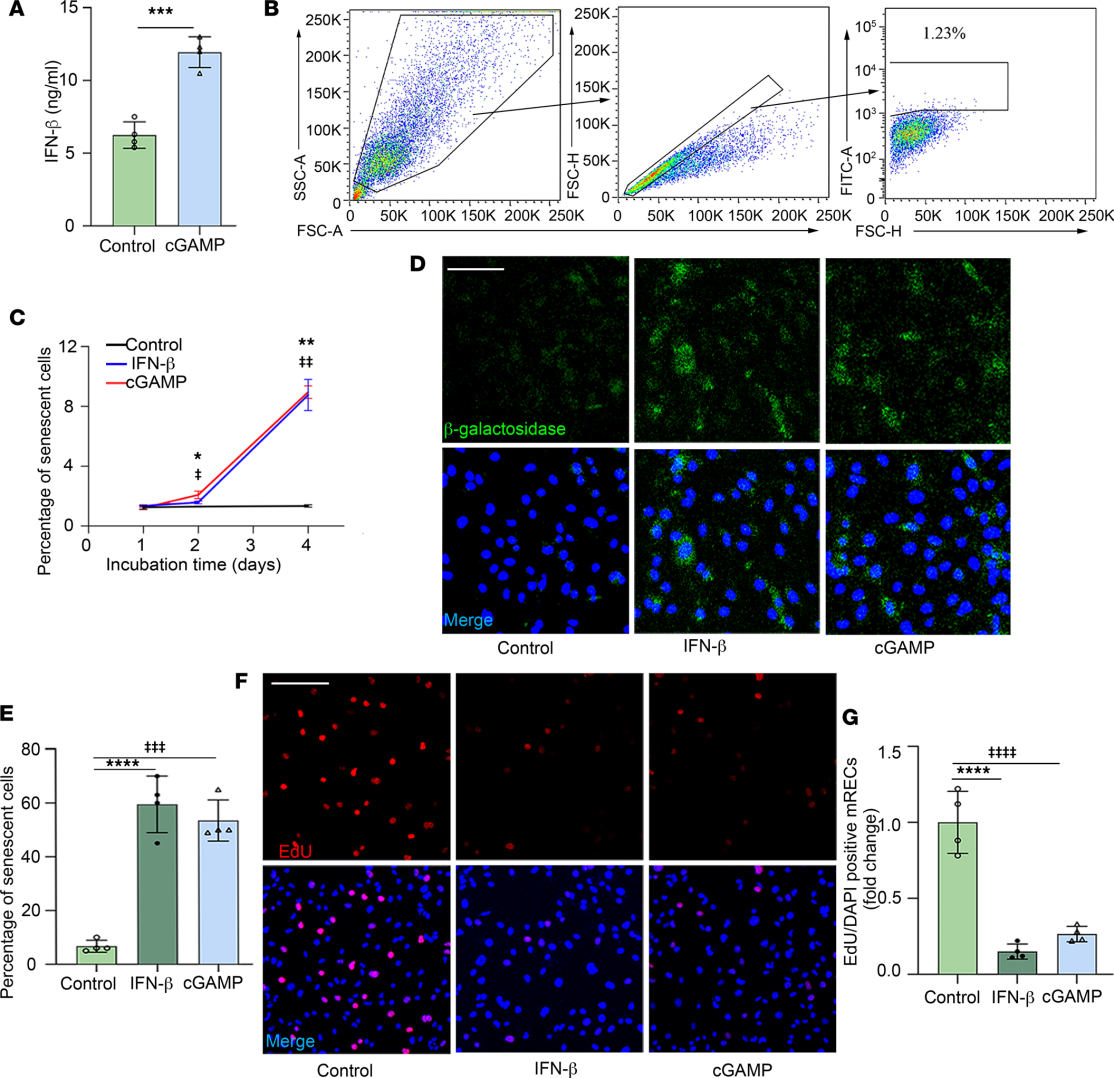
Activation of STING leads to increased secretion of IFN-β, which contributes to cellular senescence in mouse retinal endothelial cells. (**A**) The secretion of IFN-β into the medium is higher in mRECs after cGAMP treatment compared with controls. (**B**) FSC-A versus SSC-A dot plots were gated to eliminate debris, single cells were selected on FSC-A versus FSC-H, and positive senescent cells emit a fluorogenic signal that has absorption/emission maxima of 490/514 nm, which fall within the FITC detection region. (**C**) Quantification of senescent mRECs after IFN-β and cGAMP treatment for 1, 2, and 4 days. A significantly higher percentage of senescent cells was observed in the IFN-β– and cGAMP-treated groups after 2 and 4 days of incubation compared with nontreated control groups. (**D**) Representative images for immunocytochemical study. Scale bar: 50 μm. Green represents high β-galactosidase activity; blue, Hoechst. (**E**) Quantification demonstrating a higher percentage of senescent cells in the IFN-β– and cGAMP-treated groups after 4 days of treatment compared with nontreated controls. (**F**) Representative images for proliferation assay. Scale bar: 50 μm. Green, EdU; blue, Hoechst. (**G**) IFN-β– and cGAMP-treated cells exhibit lower rates of proliferation than the control, which was partially rescued by the STING inhibitor. Values are the mean of 4 replicates ± SD. *P < 0.05, **P < 0.01, ***P < 0.001, ****P < 0.0001 for the effects of IFN-β versus control groups. ‡‡‡*P* < 0.001, ‡‡‡‡*P* < 0.0001 for the effects of cGAMP versus nontreated control. Significance was examined by Mann-Whitney *U* test (**A**) or Kruskal-Wallis test followed by Tukey’s multiple-comparison test (**C**–**G**).

Recent studies have drawn attention to the key role of cellular senescence in DR pathogenesis (8, 9, 54). In particular, endothelial cell senescence has been reported to be an early event leading to vascular injury in diabetic eyes (8). Previous studies have shown that senescent endothelial cells exhibit increased leukocyte attachment and enhanced expression of adhesion molecules such as ICAM-1 (28). Furthermore, elevated IFN-β in diabetic retinas has also been implicated in cell senescence (40). We therefore postulated that STING’s contribution to retinal leukostasis in diabetes may be mediated by enhanced expression of ICAM-1 by IFN-β–induced senescent endothelial cells. To investigate this hypothesis, we first confirmed that the secretion of IFN-β into the culture medium of mouse RECs (mRECs) was increased following activation of STING by cGAMP compared with untreated controls (Figure 6A). We further conducted a retinal explant experiment (55) and found the levels of senescence-associated secretory phenotype (SASP) mediators, like HMGB1 and IL-1β, were high in the spent media from high glucose–exposed (HG-exposed, 25 mM D-glucose) WT retinal explants, compared with WT retinal explants cultured in presence of low glucose (LG, 5 mM D-glucose). However, HG-exposed *STING*-KO and *STING*^GT^ retinal explants did not show such changes, indicating that STING might be involved in retinal cell senescence in diabetes (Supplemental Figure 4).

To examine whether this increase in IFN-β could promote an increase in endothelial cell senescence, we incubated mRECs with IFN-β or cGAMP for 1 to 4 days, and then compared them to control mRECs. Using the CellEvent Senescence Green Flow Cytometry Assay, we observed a significantly higher percentage of senescent cells (green color) in the IFN-β– and cGAMP-treated mRECs after 2 days of incubation compared with nontreated groups; this increase was sustained at 4 days (Figure 6, B and C). These results were corroborated by immunocytochemistry (Figure 6, D and E). Furthermore, we observed that IFN-β– and cGAMP-treated cells exhibited much lower rates of proliferation than control cells (Figure 6, F and G).

**Figure 7:**
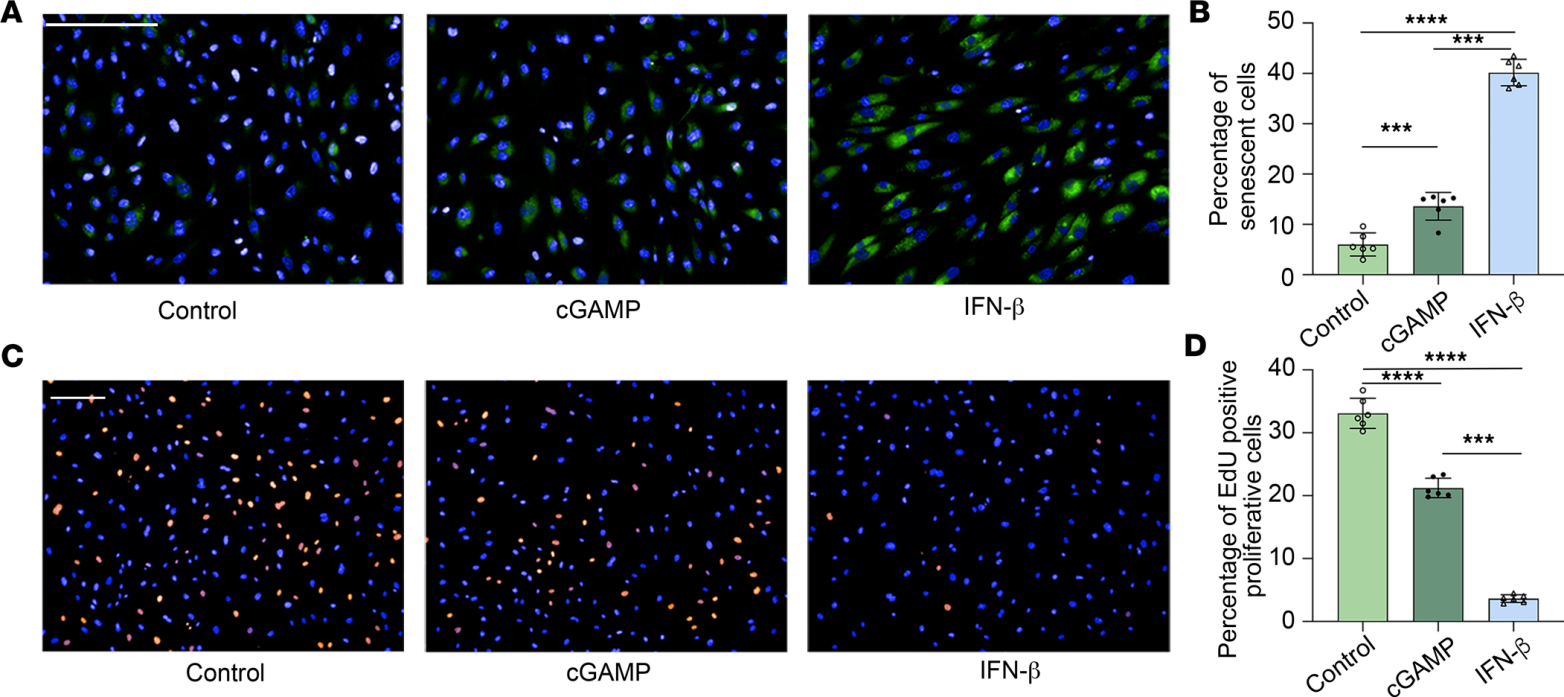
cGAMP and IFN-β induce cellular senescence and reduced cell proliferation in human retinal endothelial cells. (**A**) Representative images for β-galactosidase assay. Original magnification, ×20; scale bar: 50 μm. (**B**) Quantification demonstrating a higher percentage of senescent cells in the IFN-β– and cGAMP-treated groups. (**C**) Representative images for EdU assay. Original magnification, ×10; scale bar: 50 μm. (**D**) IFN-β– and cGAMP-treated cells exhibit lower rates of proliferation than control. Green, β-galactosidase activity; blue, Hoechst; red, EdU. Percentage of EdU-positive senescent cells and percentage of EdU-positive proliferating cells are expressed as mean ± SD (*n* = 6). ***P < 0.001; ****P < 0.0001 by Kruskal-Wallis test followed by Tukey’s multiple-comparison test.

A similar effect was also observed when human RECs (hRECs) were treated with IFN-β and cGAMP. The percentages of senescent cells in INF-β– and cGAMP-treated groups were significantly increased compared with nontreated control cells (Figure 7, A and B). Cell proliferation was also inhibited by INF-β and cGAMP treatment, as more nonproliferative cells were present in these 2 groups compared with the control group (Figure 7, C and D). Taken together, these data suggest that STING activation regulates endothelial cell senescence in both mice and humans through upregulation of INF-β.

### STING contributes to diabetes-induced retinal vascular cell injury through the STING/TBK1/IRF3/IFN-β signaling pathway.

**Figure 8:**
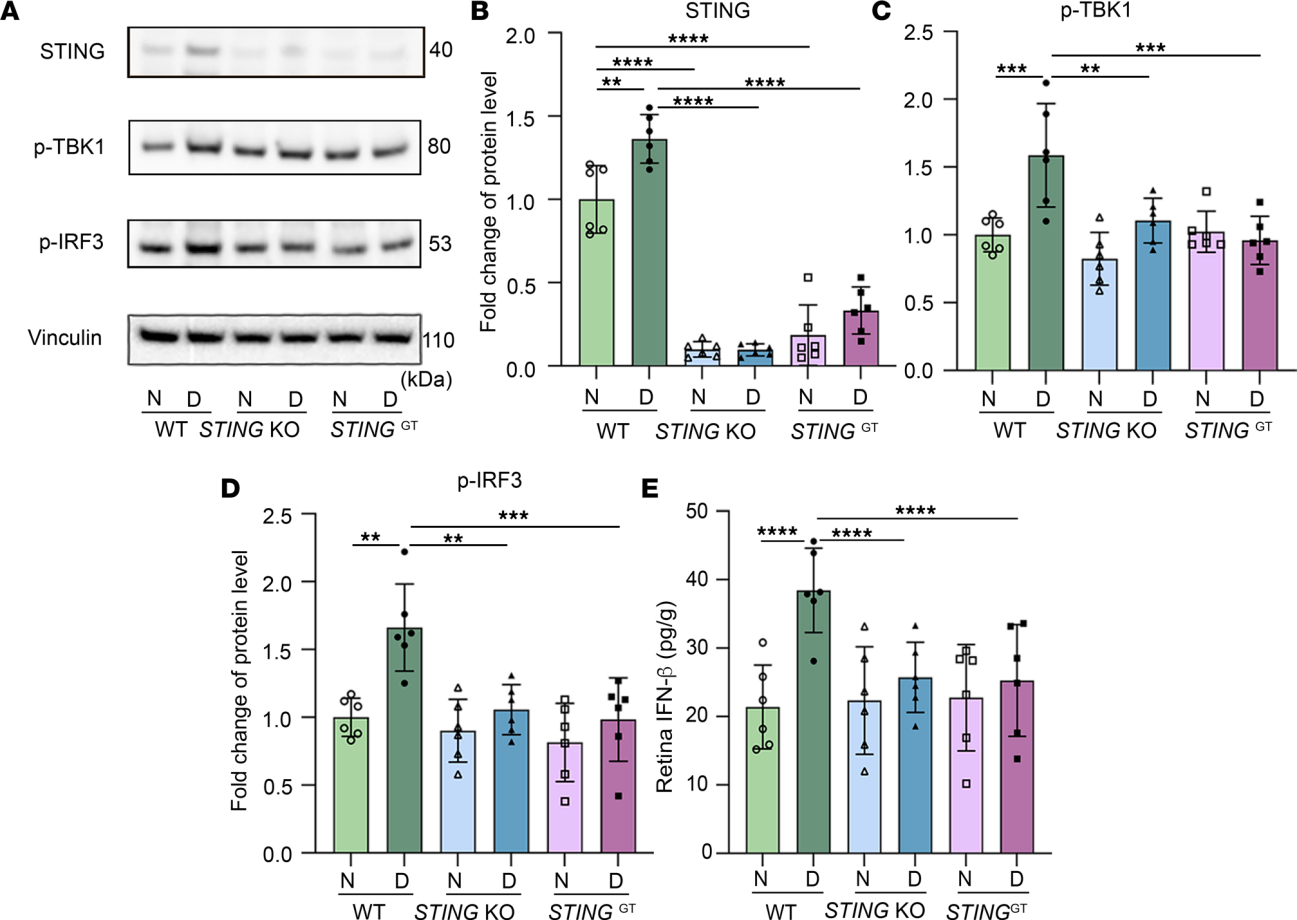
Blocking STING inhibits diabetes-induced increases in IFN-β in the retina through STING/TBK1/IRF3 signaling. (**A**) Representative immunoblots and quantification of (**B**) STING, (**C**) p-TBK1, and (**D**) IRF3. Induction of diabetes in WT mice resulted in increased STING compared with appropriate controls; STING was not expressed in *STING*-KO and *STING*^GT^ mice. p-TBK1 and p-IRF3 were decreased in *STING*-KO and *STING*^GT^ diabetic mice compared with WT diabetic controls. ELISA analysis (**E**) shows that IFN-β was increased in WT diabetic retina compared with nondiabetic controls, but this was inhibited in *STING*-KO and *STING*^GT^ diabetic mice. *n* = 6 mice for each group; the data are expressed as mean ± SD. Statistical differences were examined by ordinary 1-way ANOVA followed by Tukey’s multiple-comparison test. **P < 0.01, ***P < 0.001, ****P < 0.0001 versus nondiabetic WT control. N, nondiabetic; D, diabetic.

To further elucidate the molecular mechanism(s) by which STING promotes diabetes-induced retinal vascular cell injury, we investigated the role of downstream effectors of the cGAS/STING pathway, including TBK1 and IRF3. TBK1 is a critical downstream effector of STING activation that recruits and activates IRF3 (56). The TBK1/IRF3 pathway has previously been implicated in the induction of type I IFNs by STING (57). We found the expression of p-IRF3 and p-TBK1 was significantly increased in the retinas of diabetic mice 2 months after the induction of diabetes, prior to the development of microvascular injury. Conversely, their expression levels were markedly diminished in *STING*-KO and *STING*^GT^ diabetic mice (Figure 8, A–D). Accordingly, IFN-β levels were also significantly lower in *STING*-KO and *STING*^GT^ diabetic mouse retinas compared with those of WT diabetic retinas (Figure 8E). Collectively, these data suggest that the activation of TBK1/IRF3 signaling may cause the accumulation of STING in diabetic retinas required for increased IFN-β expression.

### The STING/TBK1/IRF3/IFN-β signaling pathway promotes REC senescence.

**Figure 9:**
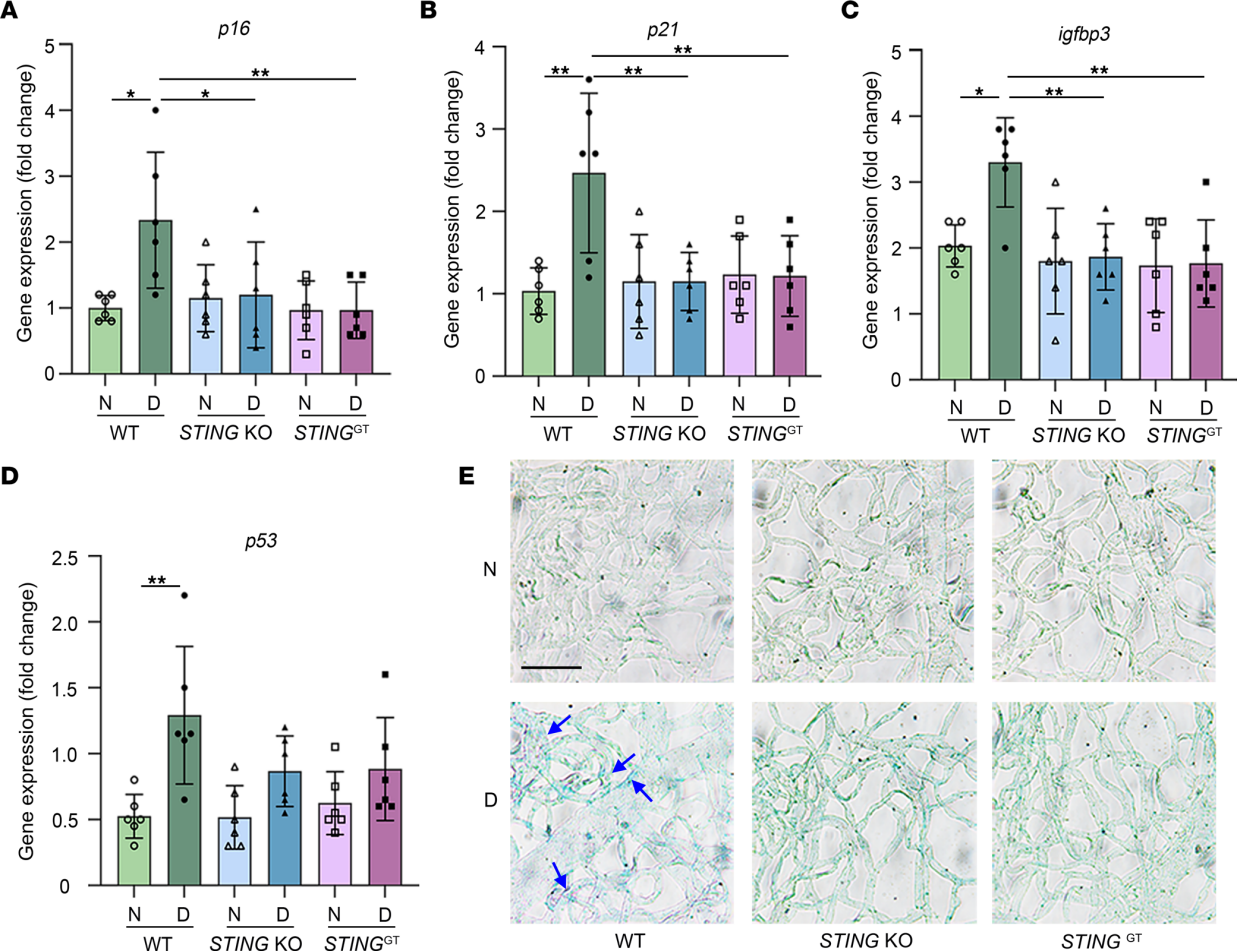
Blocking STING inhibits diabetes-induced increases in cellular senescence markers in retinal blood vessels. Diabetes increased retinal vascular mRNA levels for (**A**) *p16*, (**B**) *p21*, (**C**) *Igfbp3*, and (**D**) *p53* compared with levels in nondiabetic mice. *STING*-KO and *STING*^GT^ significantly inhibited *p16*, *p21*, and *Igfbp3*, but not *p53*, in diabetic mice. (**E**) Representative images show a general development of a blue color (β-galactosidase activity, arrows) in the freshly isolated retinal blood vessel from WT diabetic mice but not from *STING*-KO and *STING*^GT^ mice. Scale bar: 50 μm. For **A**–**D**, *n* = 6 mice for each group; the data are expressed as mean ± SD. Statistical differences were examined by ordinary 1-way ANOVA followed by Tukey’s multiple-comparison test. *P < 0.05, **P < 0.01 versus nondiabetic WT control. N, nondiabetic; D, diabetic.

Recent studies have demonstrated that endothelial cell senescence is increased in STZ diabetic mice and humans with PDR (8, 9). However, the underlying mechanism of this phenomenon remains unclear. It has been reported that type I IFNs induce p53 expression in response to DNA damage and contribute to cellular senescence (40, 58). Studies have further shown that the activation of STING by micronuclei-derived cytosolic chromatin fragments, or by the RNA-DNA hybrids in RNase H2–deficient cells, leads to cell senescence through accumulation of type I IFNs (59–62). These studies suggest that DNA damage signaling may stimulate cell senescence through type I IFN signaling. In this regard, it has been reported that senescence markers (e.g., p16^INK4a^) are increased in the retinas of patients with PDR as well as in mouse models of ischemic retinopathy (54). Interestingly, we observed that in retinal blood vessels, STING expression and the mRNA levels of cell senescence markers, including *p16*, *p21*, *Igfbp3*, and *p53*, were significantly increased in diabetic WT mice compared with nondiabetic controls (Figure 9, A–D). Conversely, the mRNA levels of these cell senescence markers were significantly decreased in the retinal vessels of *STING*-KO and *STING*^GT^ compared with WT diabetic mice (Figure 9, A–D), consistent with a role for STING in promoting REC senescence. We further observed β-galactosidase activity in the retinal blood vessels of WT diabetic mice (2-month duration of diabetes), but not in the *STING*-KO and *STING*^GT^ diabetic mice (Figure 9E), suggesting that REC senescence caused by diabetes requires STING activation. These data suggest that diabetes-induced retinal vascular lesions may be due to the activation of STING/TBK1–mediated REC senescence.

## Discussion

DR is a multifactorial disease characterized by progressive alterations in the microvasculature that lead to retinal capillary degeneration, and can result in retinal ischemia and neovascularization (7). Previous studies of DR have demonstrated that multiple biological signaling pathways in a variety of cell types in the retina may collectively contribute to the pathogenesis of this complex disease (11, 23). Despite recent advances in our understanding of DR, the early events that trigger the development of retinal vascular injury in patients with diabetes is poorly understood. Prior studies have explored the role of cellular senescence, inflammation, and oxidative stress in retinal capillary degeneration during the development of DR (8, 10–12). However, whether the cGAS/STING pathway, recently implicated in retinal microvascular disease (63, 64), could contribute to these processes has remained unclear. In this study, we provide evidence suggesting that the cGAS/STING pathway is activated in the retinas of diabetic human donors as well as in the retinas of diabetic mice, and that this pathway promotes an increase in the secretion of IFN-β, which in turn contributes to endothelial cell senescence. Using 2 independent genetic mouse models, we further demonstrate that the inhibition of STING significantly inhibits diabetes-induced REC senescence, while decreasing the expression of retinal inflammatory proteins, production of ROS, pericyte loss, and capillary degeneration in diabetic mice. Collectively, these observations suggest that STING might be a novel therapeutic target for DR.

A role for STING in cellular senescence and inflammation has recently been reported (33). Prior reports demonstrating STING-enhanced senescence in chondrocytes exposed to IL-1β (65) and HG-induced increased β-galactosidase activity in cultured endothelial cells (8) support a role for STING in REC senescence in diabetes. However, the underlying mechanism(s) linking STING to DR is not known. Recent evidence shows that cGAS catalytic activity triggered through interactions with double-stranded DNA leads to the activation of STING, which can regulate cellular senescence, inflammation, and oxidative stress, processes that are central to the development and progression of DR (33). Here we provide evidence that increased expression of STING in the retina of human DR donors is associated with an increased level of free DNA in patients with DR, suggesting that the activation of cGAS/STING signaling in diabetes may be due to the accumulation of free DNA in the eye.

It has been previously reported that the activation of STING leads to cellular senescence by activating the kinase TBK1, which in turn phosphorylates the transcription factor IRF3 and contributes to the production of type I IFNs (37–39). Type I IFNs lead to cell senescence in endothelial cells and have been implicated in DR (40). We provide evidence that elevated levels of IFN-β are present in the retinas of both human DR donors and diabetic mice and that genetic inhibition of STING abolished diabetes-induced secretion of IFN-β and REC senescence; this correlated with a reduction in the levels of p-TBK1 and p-IRF3. Collectively, these results suggest that diabetes-induced REC senescence may be due to the activation of STING and the subsequent increase IFN-β through TBK1/IRF3 signaling.

STING is a multifunctional protein that is not only involved in type I IFNs secretion but also regulates inflammation, thereby contributing to the process of cell senescence (24–26, 33). Activation of STING, through TBK1 and IκB kinase epsilon (IKKε), has been reported to activate the IKK complex, triggering NF-κB–regulated secretion of proinflammatory cytokines (51, 66) and type I IFNs (67). Interestingly, we found that genetic inhibition of STING attenuates diabetes-induced phosphorylation of TBK1, IKK, IκB, and NF-κB, which is associated with reduced iNOS and ICAM-1 expression along with decreased superoxide production and leukostasis in STING-perturbed diabetic mice. Inflammation and oxidative stress contribute to the retinal capillary degeneration in diabetes and have been reported to promote cellular senescence (24–26, 68). Activation of the STING/TBK1/NF-κB inflammatory pathway may also contribute to the REC senescence, providing a link between STING and the development of DR through NF-κB–regulated inflammation and cell senescence. Of note, it has previously been reported that the presence of senescent endothelial cells is associated with increased ICM-1 expression, which, in turn, increases leukocyte adhesion (25). We postulate that the increased leukostasis in the diabetic retina may be mediated through increased expression of ICAM-1 in RECs by STING-regulated endothelial cell senescence.

**Figure 10:**
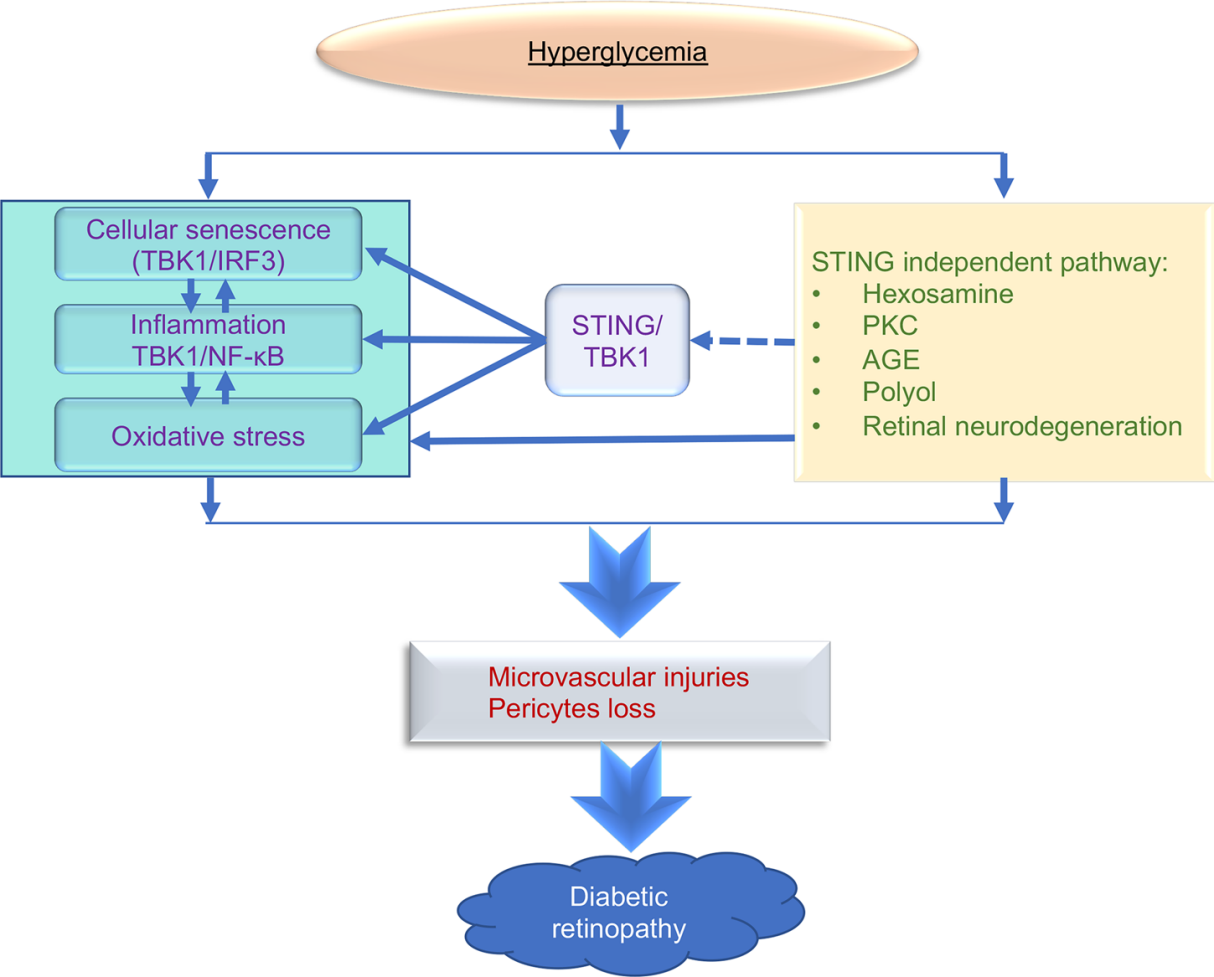
A schematic illustration of the role of STING in diabetic retinopathy (DR). STING-mediated induction of the retinal type I IFNs secretion through TBK1/IRF3 and inflammatory molecules via the TBK1/NF-κB pathway in diabetes results in increased endothelial cell senescence and production of superoxide. These factors combine to cause retinal microvascular injuries in DR. Hyperglycemia evokes various other pathological mechanisms such as retinal neurodegeneration, accumulation of AGEs, and induction of PKC, the polyol, and the hexosamine pathways also critical in DR, independently of STING. STING may serve as a hub that stimulates multiple pathways (also promoted by these other factors) that collectively promote this multifaceted disease. All processes are interconnected to the development and progression of DR. AGEs, advanced glycation end products; PKC, protein kinase C; ROS, reactive oxygen species.

While previous studies have confirmed that multiple signaling pathways may contribute to the pathogenesis of DR, our results provide evidence for a central role for STING in potentiating multiple pathways, including inflammation, oxidative stress, and cellular senescence, all critical factors that drive early DR pathogenesis. Nonetheless, other factors induced by hyperglycemia, including accumulation of advanced glycation end products (AGEs), lipid metabolic abnormalities, and neuronal degeneration, are also pivotal for DR pathogenesis (69), but are independent of STING. Thus, activation of STING, alone, is not likely to be sufficient to promote the vascular cell injury observed in DR (Figure 10). Rather, inflammation and oxidative stress may combine with several pathways such as induction of protein kinase C (PKC), and activation of the polyol and hexosamine pathways to damage the diabetic retina more severely after induction of these pathways. Nevertheless, our data suggest that diabetes-induced retinal capillary lesions may occur through STING/TBK1–regulated REC senescence through the IRF3/IFN-β and NF-κB pathways, making STING pivotal to connecting multiple signaling pathways that contribute to DR pathogenesis (Figure 10). Thus, STING is an important early target for therapeutic intervention.

## Methods

### Animals and diabetes.

WT C57BL/6J (strain 000664), *STING*-KO, strain 025805), and *STING*^GT^ (strain 017537) mice were purchased from The Jackson Laboratory and were housed in ventilated microisolator cages in 12-hour light/dark cycles. *STING*-KO mice lack exons 3–5 of the transmembrane protein 173 (*Tmem173*) gene, and Golden Ticket (*Tmem173*^GT^) is an I199N missense mutant allele of the *STING* gene, which does not produce IFN-β in response to cyclic dinucleotides or *Listeria monocytogenes* infection, as indicated on The Jackson Laboratory website. At 2 months of age, mice were randomly selected from each strain. Insulin-deficient diabetes was induced in these mice by i.p. injection of STZ (S0130, Sigma-Aldrich) for 5 consecutive days at a concentration of 60 mg/kg (BW) and insulin (0–0.2 units every 2–3 days; 12585014, Invitrogen) was administered as needed to prevent weight loss while still allowing chronic hyperglycemia. In addition, fasting blood glucose was measured as previously reported (3). The onset of diabetes was determined when blood glucose readings exceeded 275 mg/dL consistently on 3 different days. HbA1c was measured using the Mouse HbA1c Assay Kit (80310, Crystal Chem) and its control (80313, Crystal Chem) when the mice were euthanized to assess the severity of hyperglycemia. Clinical data from these diabetic mice are shown in Supplemental Table 4.

### Western blot analysis.

Retinal homogenates were prepared as previously described (4). The retinas were placed into RIPA lysis buffer (20-188, Millipore) containing 1% phosphatase (P0044, Sigma-Aldrich) and 1% protease (I3786, Sigma-Aldrich) inhibitors. Samples were sonicated and then centrifuged at 14,000*g* for 20 minutes at 4°C. The protein concentrations were estimated using a BCA assay (23225, Thermo Fisher Scientific) and measured by a microplate reader (BioTek Synergy H1 Hybrid reader) at an absorbance of 562 nm. The samples were then mixed with sample buffer (NP0007, Life Technologies) containing 10% 2-mercaptoethanol (M3148, Sigma-Aldrich) and boiled for 10 minutes. The proteins were separated using 4%–12% Bis-Tris Nu-PAGE gels (NW04125BOX, Thermo Fisher Scientific) and were transferred to nitrocellulose membranes for immunoblotting. After being blocked with 5% milk (170-6404, Bio-Rad) for 1 hour, the membranes were incubated overnight with primary antibodies (diluted 1:1000) against iNOS (2982S, Cell Signaling Technology), ICAM-1 (10020-1-AP, Proteintech), p-IκBα (Ser32) (2859T, Cell Signaling Technology), IκBα (4812S, Cell Signaling Technology), STING (NBP2-24683, Novus Biologicals), cGAS (15102S, Cell Signaling Technology), p-TBK1 (5483S, Cell Signaling Technology), TBK1 (3013S, Cell Signaling Technology), p-IRF3 (29047S, Cell Signaling Technology), IRF3 (4302S, Cell Signaling Technology), p-NF-κB (3033S, Cell Signaling Technology), NF-κB (6956S, Cell Signaling Technology), GAPDH (5174S, Cell Signaling Technology), or vinculin (ab129002, Abcam). After primary antibody incubation, the membranes were washed with TBS/0.1% Tween 20 (351-086-131, Thermo Fisher Scientific; P7949, Sigma-Aldrich respectively) and incubated with goat anti-rabbit secondary antibody (1:2000; 5220-0336, SeraCare) in 5% milk (w/v) at room temperature for 1 hour. Then, the membranes were washed 3 times in TBS/0.1% Tween 20 for 10 minutes. Finally, the membranes were developed using the ECL Western Blotting Detection Reagent (RPN2209, GE Healthcare), and images were acquired using the Azure c400 system.

### Immunohistochemical staining.

Immunohistochemical detection of STING in human eyes employed a Ventana Discovery Ultra automated staining system with Discovery CC1 antigen retrieval (Ventana-Roche Diagnostics) for 32 minutes at 95°C with an Invitrogen mouse monoclonal antibody (diluted 1:100) against full-length human recombinant TMEM173 protein produced in HEK293T cells (clone OTI4H1; MA5-26030, Thermo Fisher Scientific), Discovery OmniMap anti-mouse HRP detection system, and the Discovery Purple Kit (Ventana-Roche Diagnostics). Negative controls employed a cocktail of mouse IgG isotypes (IR750, Agilent Dako Technologies). Immunohistochemical detection of IFN-β in human eyes employed a Ventana Discovery Ultra automated staining system with Discovery CC1 antigen retrieval for 32 minutes at 95°C with rabbit anti–human IFN-β antibody (1:200; ab140211, Abcam; immunogen was a recombinant fragment corresponding to amino acids 23–187 of human IFN-β), Discovery OmniMap anti-rabbit HRP detection system, and the Discovery Purple Kit. Negative controls employed Dako FLEX Rabbit Universal Negative Control (IS60061-2, Agilent Dako Technologies). Eyes with proliferative DR were selected from autopsies performed prior to December 31, 2004 to exclude patients who had undergone intravitreal injections of anti-VEGF drugs. A few eyes were from autopsies done after January 1, 2005, but the patients had their eye care at Duke University and never received anti-VEGF drugs. Final selection of eyes were those patients with the shortest postmortem interval.

### Measurement of soluble factors and quantification of DNA in human samples.

Participants were recruited for the study after obtaining informed written consent as per institutional and ethics board guidelines and as referred to Narayana Nethralaya Eye Hospital, Bangalore, India. The inclusion and exclusion criteria for recruitment of participants for the study were as follows. Inclusion criteria: Controls – Aqueous humor (i) Participants undergoing surgical intervention (cataract surgery part of standard of care) that would require access to the aqueous humor. Controls – Vitreous humor (i) Participants with no sign of fibrovascular proliferation but other retinal conditions such as floaters and macular hole required surgical intervention (as a part of standard care) that would require access to the vitreous humor. DR – Aqueous humor (i) DR participants of varying severity diagnosed based on fundus imaging using optical coherence tomography (OCT) and fundus fluorescein angiography (FFA); (ii) Participants undergoing surgical intervention (intravitreal injections of anti-VEGF or steroids as part of standard of care) that would require access to the aqueous humor. Vitreous humor (i) Patients with DR diagnosed based on fundus imaging, OCT, and FFA. Exclusion criteria for aqueous humor and vitreous humor: (i) Participants with any other coexisting retinal pathology such as inherited retinal disorders, age-related macular degeneration, glaucoma, or infective conditions such as uveitis. Sample collection: Aqueous humor was collected by anterior chamber paracentesis where a 30-gauge needle was used to access the anterior chamber through the peripheral cornea and a volume of 50–100 μL was aspirated. Vitreous humor was collected by vitreoretinal surgeon at the beginning of 3-port pars plana vitrectomy as per standard-of-care procedure. Samples were stored at –80°C for not more than 11 months before further processing. Measurement of soluble factors: IFN-β concentration in aqueous humor samples was measured using sandwich ELISA (SEA222Hu, Cloud Clone Corp.) as per the manufacturer’s instructions. IFN-β concentration in vitreous humor samples was measured using multiplex ELISA (BioLegend LEGENDplex). Other soluble factor concentrations, including VEGF, IL-8, MCP-1, and IFN-α were measured using bead-based multiplex ELISA (Cytometric Bead Array, BDTM CBA Human Soluble Protein Flex Set System, BD Biosciences) using a flow cytometer (FACSCanto II, BD Biosciences). The required volume of aqueous humor and vitreous humor was diluted using ice-cold 1× PBS. The beads and fluorescence signal intensities were acquired and recorded using FACSDiva software (BD Biosciences). Standards were used to determine the absolute concentration of the analytes and the calculation was performed using FCAP array version 3.0 (BD Biosciences). To accommodate the variation due to vitreous humor dilution during the collection process, absolute concentrations of soluble factors were multiplied with a dilution factor calculated based on averaging the total protein concentrations of samples. Quantification of DNA: Vitreous humor samples were first separated into acellular and cellular contents by a short centrifugation cycle at 400*g* and 4°C for 5 minutes. The acellular material was separated and collected in a microcentrifuge tube. From each sample, 100 μL was used for nucleic acid precipitation by using 3 M sodium acetate (pH 5.2) and 2.5 volumes of absolute ethanol. The samples were mixed and incubated at –80°C for 2 hours, after which samples were centrifuged at 15,000*g* for 15 minutes at 4°C. The supernatant was separated carefully without disturbing the pellet. The pellet was washed twice with ice-cold 70% ethanol and spun down at 15,000*g* for 10 minutes each at 4°C. The washed pellet was air dried and dissolved completely in nuclease-free water. The samples were measured spectrophotometrically for DNA content at 260 nm/280 nm; the DNA concentration is reported as μg/mL.

### ELISA.

Mouse IFN-α (42115-1, pbl Assay Science) and IFN-β (42410, pbl Assay Science) ELISA kits were used to measure the level of IFN-β in mouse retinal and primary mREC culture medium. Mice were euthanized by CO_2_ asphyxiation. The retina was freshly isolated and sonicated in a sample buffer provided in the ELISA kit with 1% phosphatase and 1% protease inhibitors. The mREC culture medium was collected 4 days after cGAMP (20 μg/mL; vac-nacga23, Invivogen) and STING inhibitor (2 μg/mL; inh-h151, Invivogen) treatment. Following the kit instructions, the absorbance of each sample was measured by a microplate reader (BioTek Synergy H1 Hybrid reader) at 450 nm. Lastly, the IFN titer of the samples was determined by plotting the optical densities using a 4-parameter fit for the standard curve in GraphPad Prism 9 (version 9.3.1). The retinal explants from WT, *STING*-KO, and *STING*^GT^ mice were cultured in presence or absence of LG or HG as explained above and in a previous report (55). The spent media from the cultures were analyzed by ELISA for HMGB1 (Novus Biologicals, NBP2-62767) and IL-1β (Invitrogen, BMS6002) using commercially available kits and following the manufacturer’s guidelines.

### Retinal cryosections and immunostaining.

Freshly dissected whole eyes were fixed in 4% paraformaldehyde (PFA) (J61899-AP, Alfa Aesar) for 2 hours and then the anterior parts were removed. The posterior eyecups were infiltrated with 5%, 10%, and 20% sucrose in PBS for 30 minutes each at room temperature, and the samples were finally infiltrated with 2:1 mixture of 20% sucrose in PBS and OCT compound (Sakura Finetek) for 45 minutes and frozen completely (1–3 minutes) on dry ice. Immunofluorescence was performed on frozen sections from the posterior eyecups as described previously (70). The sections were incubated with PBS containing 5% normal donkey serum for 30 minutes and then incubated overnight at 4°C with primary antibodies for STING (NBP2-24683, Novus Biologicals) and CD31 (11-0311-82, Thermo Fisher Scientific) diluted 1:200. The sections were washed with PBS and then incubated with 1 μg/mL DAPI (62248, Thermo Fisher Scientific) and goat anti-rabbit Alexa Fluor 555–conjugated secondary antibody (1:1000) (A21428, Thermo Fisher Scientific). Images were acquired by a Zeiss LSM 710 confocal workstation.

### Cytometric bead array.

The cytometric bead array was performed using retinal lysates of diabetic and nondiabetic WT, *STING*-KO, and *STING*^GT^ mice using a previously published method (70). The assay was performed using the LEGENDplex mouse inflammation panel (740446, BioLegend). The results were analyzed using the manufacturer’s protocols and by using the LEGENDplex Data Analysis Software Suite.

### Cell culture.

hRECs (PB-CH-160-8511, Pelo Bioscience) were maintained in EGM-2 MV medium (CC-3202, Lonza) under treatment conditions for 2 weeks. In addition to the 2 treatment conditions with 1000 U IFN-β (8499-IF-010, R&D Systems) and 33 μg/mL cGAMP (tlrl-nacga23m, InvivoGen), a control arm was performed. Meanwhile, the cells underwent 3 passages (p7–p9) and the medium was changed every other or every third day. After the last passaging step, cells were split into a 96-well plate for the readout assay.

Primary mRECs (C576065, Cell Biologics) were cultured in Complete Mouse Endothelial Cell Medium (M1168, Cell Biologics) at 37°C in an incubator containing 5% CO_2_. The medium was changed every other day until the cells were confluent for use. For the flow cytometry cell senescence assay and the ELISA experiment, mRECs were seeded in a 6-well plate at a concentration of 0.2 million cells per well. For the confocal imaging cell senescence assay, mRECs were seeded on a cell culture cover glass (NC0620709, Thermo Fisher Scientific) and laid on the bottom of a 24-well plate (3527, Corning) at 0.1 million cells per well. After 24 hours of incubation, mRECs were treated with 0.1% DMSO (control), cGAMP (20 μg/mL), STING inhibitor (2 μg/mL; inh-h151, Invivogen), or IFN-β (1000 U/mL; 12400-1, R&D Systems) for an additional 1, 2, or 4 days, respectively.

### Mouse retinal blood vessel isolation.

Retinal blood vessels were isolated as previously described (8, 71). In short, the freshly isolated retinal tissue was incubated in cold water on ice for 1 hour, followed by DNase I (100 U/mL; D4527, Sigma-Aldrich) for 30 minutes. Next, the vessels were pipetted with a cut off transfer pipet (13-711-7M, Thermo Fisher Scientific) several times up and down until the neurons and glia were removed. The retinal vessels were then fixed and flat-mounted on slides, (12-550-15, Thermo Fisher Scientific) processed for a cell senescence assay, or pooled (4 retinal vessel nets from 2 mice were pooled) together without fixation for quantitative RT-PCR.

### Retinal blood vessel senescence assay.

Retinal blood vessel senescence was measured using a senescence β-Galactosidase Staining Kit (9860s, Cell Signaling Technology). Briefly, the retinal blood vessels were fixed on a slide in the fixative solution provided in the kit for 15 minutes at room temperature. The slide was then rinsed twice with PBS and cultured in a sealed container with β-Galactosidase Staining Solution. After the slides were incubated at 37°C for 24 hours in a dry incubator without CO_2,_ they were rinsed with PBS, and images were acquired using a Zeiss LSM 710 confocal microscope.

### RNA isolation and real-time RT-PCR.

According to the manufacturer’s instructions, total RNA was extracted from freshly isolated retinal blood vessels using an Isolate II RNA Mini Kit (BIO-52072, Bioline), and cDNA was generated using a SuperScript VILO cDNA Synthesis Kit (11754-050, Invitrogen). TaqMan probes for mouse p16INK4A (Mm00494449_m1, Thermo Fisher Scientific), p21 (Mm04205640_g1, Thermo Fisher Scientific), Igfbp3 (Mm00515156_m1, Thermo Fisher Scientific), and β-actin (Mm00607939_s1, Thermo Fisher Scientific) were mixed with TaqMan Gene Expression Master Mix (4369514, Thermo Fisher Scientific), and p53 Fwd: 5′-GATATCAGCCTCGAGCTCCC-3′; p53 Rev: 5′-TCCATGCAGTGAGGTGATGG-3′. β-actin Fwd: 5’-CCTGAACCCTAAGGCCAACC-3′; and β-actin: Rev: 5’-ATGGCGTGAGGGAGAGCATA-3′ were mixed with Applied Biosystems SYBR Green Master Mix (A25742, Thermo Fisher Scientific). The mRNA expression of each target was evaluated using an Applied Biosystems QuantStudio 3 qPCR machine (A28131, Thermo Fisher Scientific). Then, the gene expression levels were normalized relative to β-actin mRNA and are reported as fold change over controls using the ΔΔCt method.

### mREC and hREC senescence assays.

Flow cytometry and fluorescence microscopy were used to quantify the senescent cells in mREC cultures treated with 0.05% DMSO (control), cGAMP (20 μg/mL), STING inhibitor (2 μg/mL), or IFN-β (1000 U/mL). The cells were washed with PBS and trypsinized to create a single-cell suspension for flow cytometry. Next, the cells were fixed in 2% PFA for 10 minutes at room temperature and washed with 1% BSA in PBS to remove the fixation solution. The cells were then resuspended in 100 μL of Working Solution provided in the CellEvent Senescence Green Flow Cytometry Assay Kit (C10841, Thermo Fisher Scientific) and incubated for 2 hours at 37°C in the absence of light and CO_2_. After incubation, the Working Solution was removed, and the cells were washed twice with 1% BSA in PBS. Finally, the cells were resuspended in 500 μL of PBS with 1% BSA, and the data were analyzed on a flow cytometer using a 488 nm laser and 530 nm filter.

For fluorescence imaging, mRECs were cultured in a cell culture cover slide in 24-well plates at 0.1 million cells per well. Each well was washed with PBS followed by 2% PFA for 10 minutes at room temperature. After being washed, the cells were incubated with Working Solution for 2 hours, as mentioned above, followed by DAPI (1:500 dilution; 62248, Thermo Fisher Scientific) for 10 minutes. Then, the cells were placed on a slide, and images were acquired using Zeiss LSM 710 confocal microscope.

For the hREC senescence assay, cells were seeded onto a 96-well plate (353219, Falcon) at a cell density of 12,500 cells/well, with 6 replicates per condition. Cells were further cultivated in the cell culture incubator for 48 hours, and the medium was changed once after 24 hours. According to the manufacturer’s instructions, the detection of β-galactosidase in the cells was determined using the CellEvent Senescence Green Detection Kit. Following the assay, nuclei were stained using Hoechst 33342 (1 μg/mL; 62249, Thermo Fisher Scientific). Fluorescence was detected with the PerkinElmer Operetta.

### Proliferation assay.

For the mREC proliferation assay, cells were cultured in a cell culture cover slide in 24-well plates at 0.1 million cells per well. After 4 days of incubation in the cell culture incubator, the proliferation assay (Click-iT EdU Imaging Kit, C10338, Invitrogen) was performed using 10 μM EdU and a 24-hour incubation time. Each well was washed with PBS followed by 3.7% PFA for 15 minutes at room temperature. The cells were incubated with 0.5% Triton X-100 for 20 minutes, and the cells were washed twice with 3% BSA in PBS and then incubated in the Click-iT reaction cocktail for 30 minutes followed by Hoechst (1:2000 dilution) for 30 minutes. The slides were then prepared for imaging using Zeiss a LSM 710 confocal microscope.

For the hREC proliferation assay, cells were seeded onto a 96-well plate (353219, Falcon) at a cell density of 6250 cells/well, with 6 replicates per condition. The proliferation assay (PK-CA724-555HTS, Promokine) was performed using 5 μM EdU and a 6-hour incubation time. All other steps were done by following the instruction leaflet. Cells were analyzed with the PerkinElmer Operetta.

### Leukostasis.

Leukostasis was assayed as previously described (3). In short, the mice were perfused with prewarmed saline buffer for 2 minutes, followed by 10 mL of diluted concanavalin A–FITC solution (1:50; FL-1001, Vector Laboratories) for 1 minute, and saline for another 2 minutes. The retina was then fixed in 4% PFA for 15 minutes at room temperature and flat-mounted on a slide. FITC-labeled leukocytes in the blood vessels of the entire retina were counted using a Zeiss LSM 710 confocal microscope.

### Retinal superoxide measurement.

Retinal superoxide was evaluated by the lucigenin (bis-*N*-methylacridinium nitrate) method (3). In brief, freshly isolated retinas were incubated in a glass culture tube (033410, Thermo Fisher Scientific) with 200 μL of Krebs-Hepes buffer containing 5 mM or 25 mM glucose (G8644, Sigma-Aldrich) for 5 minutes at 37°C in 5% CO_2_. Lucigenin (M8010, Sigma-Aldrich) was added to each sample at a final concentration of 0.54 mM, and incubated for 5 minutes. The luminescence was measured by a Glomax 20/20 luminometer (Promega). The retina was then rinsed in PBS and sonicated in RIPA buffer. The protein concentrations were measured using a BCA assay (23225, Thermo Fisher Scientific), and superoxide data are reported in arbitrary units per mg of retinal protein.

### Capillary degeneration.

Isolation of retinal capillaries has been described previously (3). Diabetic (8 months’ duration) and age-matched nondiabetic mice were anesthetized by CO_2_ asphyxiation. Their eyes were fixed in 4% PFA for 2 days and incubated in an elastase (324682, EMD Millipore) solution at a concentration of 40 U/mL at 37°C for 2 hours, followed by incubation in Tris buffer pH 8.5 overnight at room temperature. Retinal blood vessels were isolated and placed on a glass slide and dried for 24 hours. The slides were immersed in periodic acid for 8 minutes, Schiff’s reagent (SS32-500, Thermo Fisher Scientific) for 15 minutes, and stained with Harris’s hematoxylin (SH26-500D, Thermo Fisher Scientific) for 15 minutes. The slides were then dehydrated with increasing ethanol concentrations (70%, 80%, 95%, and 100% ethanol) and then xylene for 2 minutes each. Acellular capillaries were identified as capillary-sized vessel tubes lacking nuclei along their length. Six field areas corresponding to the mid-retina were counted per retina to determine the number of acellular capillaries per square millimeter of retinal area.

### Permeability assay.

The leakage of albumin into the neural retina was conducted in mice by tail vein injection of sterile FITC-BSA (A9771, Sigma-Aldrich) at a dose of 200 μg/g body weight and allowed to circulate for 2 hours. The plasma was collected before animals were perfused with warm saline through the heart for 2 minutes. The retinas were then harvested and dried overnight to obtain dry weights. FITC-BSA was extracted from retinal tissues using 1% Triton X-100 in PBS. The retinal FITC-BSA value was normalized to the plasma FITC-BSA concentration, retinal dry weight, and time of dye circulation.

### Statistics.

For human aqueous humor and vitreous humor samples data analysis, Shapiro-Wilk normality test was performed to determine the distribution of the data, and the Mann-Whitney *U* test and Kruskal-Wallis test followed by Tukey’s multiple comparisons test were used to determine the difference in parameters between the test groups. Statistical analysis was performed with GraphPad Prism 6.0 and data are expressed as mean ± SEM. Statistical analysis for the rest of the study was performed using GraphPad 9.0 software and Excel (Microsoft, version 16.54); data are expressed as mean ± SD, and 2-tailed, unpaired Student’s *t* test was used to evaluate the difference whenever only 2 groups were compared in a data set. One-way ANOVA followed by Tukey’s post hoc test was used for multiple-group comparisons to evaluate the significant of the differences for the rest of the data. *P* less than 0.05 was considered significant.

### Study approval.

All animal experiments followed the guidelines set forth by the NIH *Guide for the Care and Use of Laboratory Animals* (National Academies Press, 2011) and were authorized by the University of Pittsburgh Institutional Animal Care and Use Committee. Participants were recruited for human retinal soluble factor and DNA measurements in the study after obtaining informed written consent as per institutional and ethics board guidelines and as referred to Narayana Nethralaya Eye Hospital, Bangalore, India. The human retinal section immunohistochemical staining study was deemed exempt from Institutional Review Board purview, as Title 45, Code of Federal Regulations, Part 46 (Protection of Human Subjects) defines a human subject as a living individual. The tenets of the Declaration of Helsinki and the regulations of the Health Insurance Portability and Accountability Act were followed.

### Data availability.

This study includes no data deposited in external repositories. All data generated or analyzed during this study are included in the article and its Supporting data XLS file. Complete, unedited blots can be obtained upon request from the corresponding authors.

## Author contributions

DS and DF designed the study and supervised the work. HL, SG, SB, APN, CH, TV, SS, ADP, and AG conducted the experiments and/or analyzed the data. PEF provided some of the human nondiabetic and diabetic (diabetic patients with DR) donor cadaver eye tissues. AG collected aqueous humor and vitreous humor samples. HL, SG, P Shang, OC, NAS, SH, DF, and DS wrote the manuscript. A Sodhi, HL, SGG, TMB, EML, JSZ, P Strassburger, GW, PEF, PDW, ADP, NM, A Strizhakova, JAS, DF, and DS reviewed the data and revised the manuscript.

## Supplementary Material

Supplemental data

Supporting data values

## Version 1 06/22/2023

Electronic publication
