# Supplementary material for: Activated cGAS/STING signaling elicits endothelial cell senescence in early diabetic retinopathy
Source: JCI Insight. 2023 Jun 22;8(12):e168945. doi: 10.1172/jci.insight.168945 (PMC10371250; doi:10.1172/jci.insight.168945)
Supplement: Supplemental data [file jciinsight-8-168945-s083.pdf]

Supplementary Materials for

Activated cGAS-STING signaling elicits endothelial cell senescence in early diabetic retinopathy

Haitao Liu et al.

Corresponding author: Debasish Sinha, Debasish@pitt.edu; Derrick Feenstra, derrick.feenstra@roche.com; Arkasubhra Ghosh, arkasubhra@narayananethralaya.com

This PDF file includes:
Supplemental Figures 1 - 4
Supplemental Tables 1 - 4

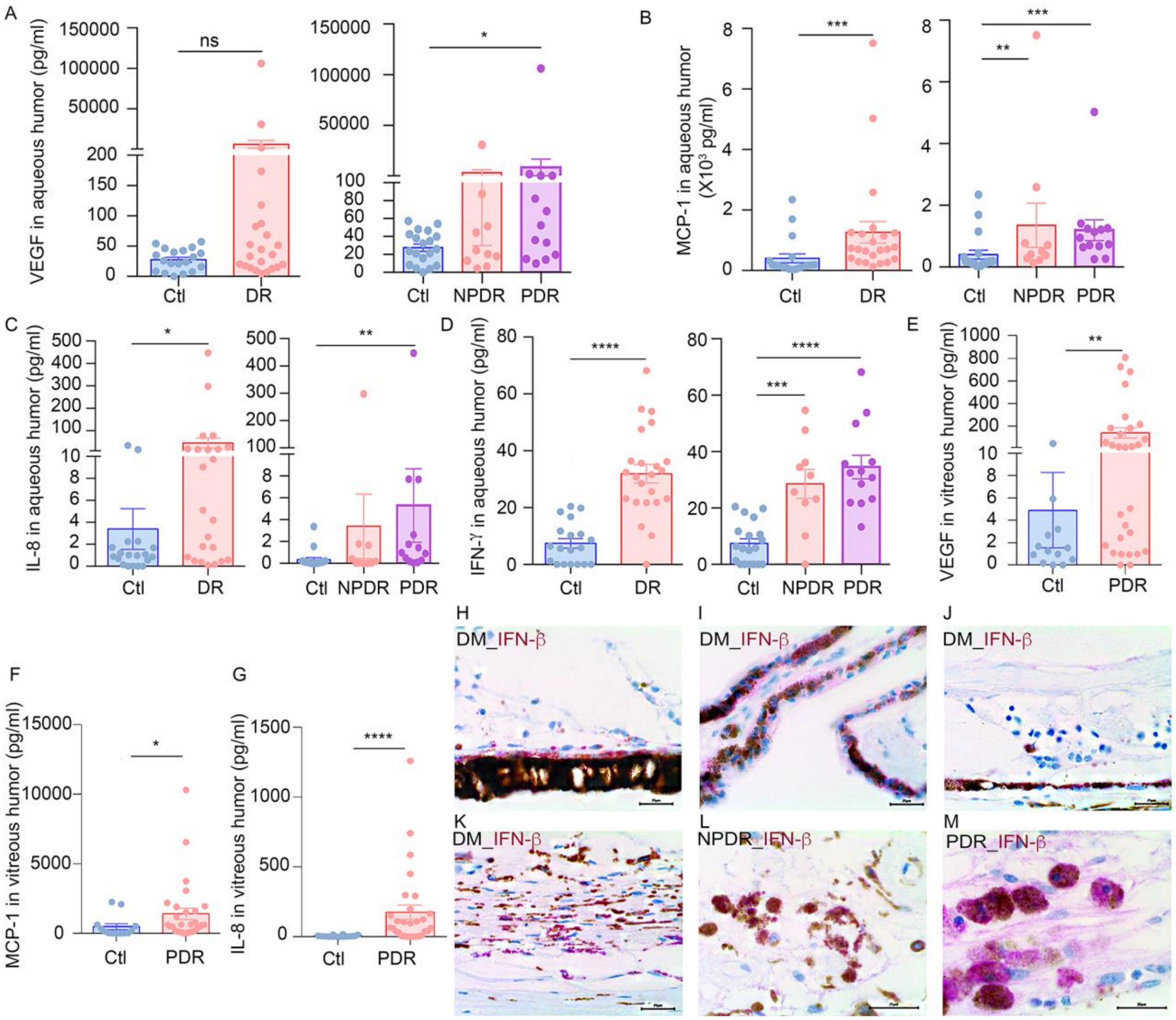

Supplemental Figure 1 – Elevated VEGF, MCP-1, IL-8, IFN-γ and IFN-β immunostaining in patients with diabetic retinopathy. VEGF (**A**); MCP-1 (**B**); IL-8 (**C**) and IFN-γ (**D**) in the aqueous humor, VEGF (**E**); MCP-1 (**F**); and IL-8 (**G**) in the vitreous humor of patients without (Ctl) and with DR or PDR. (Ctl, n = 20, DR, *n* = 23; NPDR, *n* = 10; PDR, *n* = 13 for A-D; Ctl, n = 13, PDR, *n* = 30 for E-G). Immunostaining of eyes with DR showed IFN-β expression in cells of the iris anterior epithelial layer/dilator pupillae muscle, patchy staining of the iris anterior border layer (**H**), pigmented epithelium of the ciliary body pars plicata (**I**), pars plana, peripheral retinal pigment epithelium in areas of ischemic atrophy and cystoid degeneration (**J**), and choroidal macrophages and melanocytes (**K**). **H**-**K**: DM, no histological diabetic eye disease. NPDR and PDR eyes had IFN-β staining of iris stromal clump cells (**L**). Macrophages and/or melanophages expressing IFN-β were present in the neurosensory retina (**M**). Data represent mean ± SEM. * $p < 0.05$, ** $p < 0.01$, *** $p < 0.001$, and **** $p < 0.0001$; Mann-Whitney U test and Kruskal-Wallis test. DM – diabetes mellitus; Ctl – control; NPDR – non-proliferative diabetic retinopathy; PDR – proliferative diabetic retinopathy.

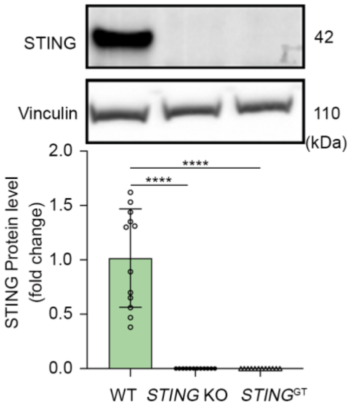

Supplemental Figure 2 – Verification of STING protein loss in KO mice. (**A**) Representative immunoblots and densitometry graphs demonstrate STING is not detectable in *STING* KO and *STING* ^{GT} mice compared to the WT group. The sample used here is leukocytes isolated from WT, *STING* KO, and *STING* ^{GT} mice. Non-diabetic and diabetic samples from the same genotype were pooled together. Data represent mean ± SD. Statistical differences were examined by ordinary one-way ANOVA followed by Tukey's multiple comparisons test, **** $p < 0.0001$. KO, knock out.

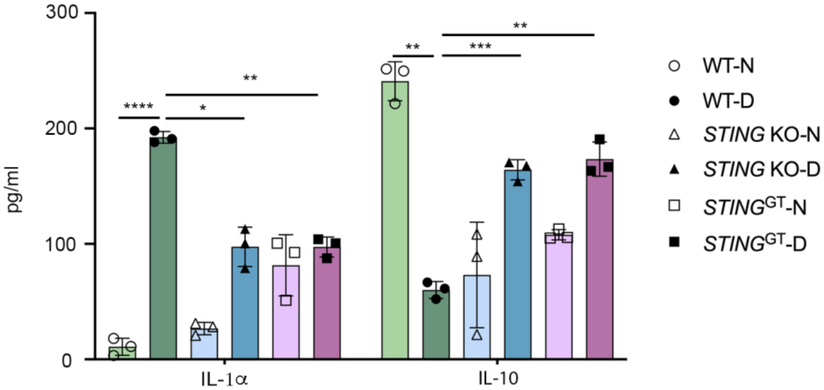

Supplemental Figure 3 – Targeting STING rescues diabetic-induced upregulation of proinflammatory mediators in the retina. Cytometric bead array (CBA) showing elevated levels of pro-inflammatory cytokines IL-1α along with reduced levels of anti-inflammatory cytokine IL-10 in retinal lysates from STZ-injected (diabetic; D) WT animals, compared to controls (non-diabetic WT; WT N). These changes were rescued in diabetic (D) *STING* KO and *STING*^{GT} retina. Data represent mean ± SD. Two-way ANOVA followed by Tukey's multiple comparisons test, *P<0.05, **P<0.01, ***P<0.001, ****P<0.0001. n=3.

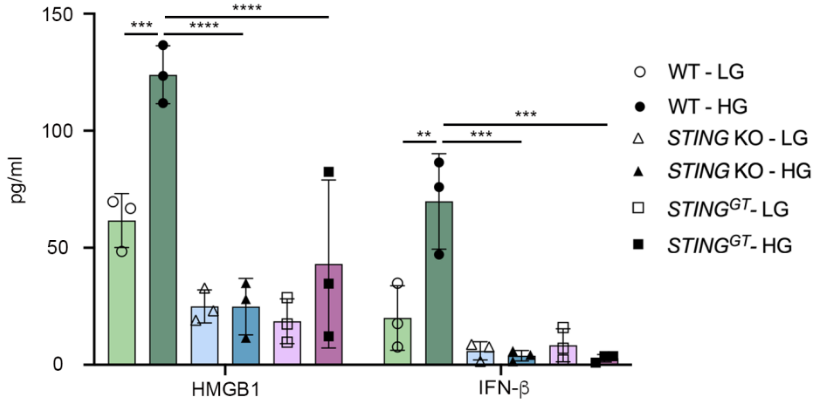

Supplemental Figure 4 – Genetic ablation of *STING* rescues diabetic-induced release of Senescence-associated secretory phenotype (SASP) mediators from retinal explants *in vitro*. Enzyme-linked immunosorbent assay (ELISA) showing elevated levels of SASP mediators like HMGB1 and IL-1β in the spent medium from HG (high glucose)-exposed WT retinal explants, compared to WT retinal explants, cultured in presence of low glucose (LG). But HG-exposed *STING* KO and *STING*^{GT} retinal explants did not show such changes. Data represent mean ± SD. Two-way ANOVA followed by Tukey's multiple comparisons test, **P<0.01, ***P<0.001, ****P<0.0001. n=3.

Supplemental Table 1 – Cohort characteristics for human retina tissue western-blot profile

Group	Age (years) Average	Male (Number)	Female (Number)	Duration of Diabetes (Years)	HbA1c (%)	Race
ND	71 ± 7	4	2	-	-	Caucasian
DR	72 ± 9	4	2	10 ± 5.6	7.2 + 1.2	Caucasian
Data are expressed as mean ± SD. *n* = 6 for the non-diabetic and diabetic retinopathy groups. Duration of diabetes and HbA1c data is not accessible for one female case in the DR group. ND, Non-diabetic; DR, Diabetic retinopathy.						

Supplemental Table 2 – Cohort characteristics for aqueous humor soluble factor profile

Category/parameter	Control	DR	NDR	PDR
Average Age (years)	67.25±2.08	60.39±1.98**	60.40±2.27*	60.38±2.06*
Gender Ratio (M/F)	12/8	20/3	9/1	11/2
Visual acuity (Log Mar)	0.66±0.14	0.71±0.18	0.45±0.08	0.91±0.22
Random blood sugar	127.35±10.76	210.88±17.64***	171.75±17.20*	232±21.08***
Data are expressed as mean ± SEM. *n* = 14 for the control group, *n* = 23 for the diabetic retinopathy group, *n* = 10 for the non-proliferative diabetic retinopathy group, *n* = 13 for proliferative diabetic retinopathy group. DR, Diabetic retinopathy; NDR, non-proliferative diabetic retinopathy; PDR, proliferative diabetic retinopathy; M, male; F, female. *$p < 0.05$, ***$p < 0.001$ compared with control. Mann-Whitney U test.				

Supplemental Table 3 – Cohort characteristics for vitreous humor soluble factor and DNA concentrations profile

Category/parameter	Soluble factors study		DNA concentrations	
	Control	DR	Control	DR
Average Age (years)	62.07±2.60	60.39±1.98	62.91±3.01	54.59±2.79*
Gender Ratio (M/F)	7/7	21/9	5/7	12/5
Visual acuity (Log Mar)	0.75±0.16	1.45±0.13**	0.88±0.16	1.38±0.19
Random blood sugar	101.94±6.13	149.72±10.61*	102.07±7.07	154.27±15.66
Data are expressed as mean ± SEM. *n* = 14 for the control group, and *n* = 30 for diabetic retinopathy group in soluble factors study; *n* = 12 for the control group, and *n* = 17 for diabetic retinopathy group in DNA concentrations study. DR, Diabetic retinopathy; M, male; F, female. *$p < 0.05$, **$p < 0.01$, ***$p < 0.001$ compared with appropriate control. Mann-Whitney U test.				

Supplemental Table 4 – Clinical data of non-diabetic (N) and diabetic mice (D) in long-term and short-term studies.

Clinical data of non-diabetic (N) and diabetic mice (D) in long-term and short-term studies				
	Group	Body weight	HbA_{1c}	
		(g)	%	mmol/mol
Short-term studies	WT-N	33 ± 3	3.1 ± 0.1	10 ± 1.0
(2-3 months duration)	WT-D	25 ± 2*	8.3 ± 0.2*	68 ± 1.7*
	STING KO - N	32 ± 2	3.1 ± 0.1	10 ± 0.8
	STING KO - D	26± 2*	8.1 ± 0.3*	65 ± 3.6*
	$STING^{Gt}$ - N	33 ± 2	3.1 ± 0.1	10 ± 0.9
	$STING^{Gt}$ - D	26 ± 2*	8.3 ± 0.3*	67 ± 2.7*
Long-term studies	WT-N	43 ± 5	3.1 ± 0.1	10 ± 0.9
(8 months duration)	WT-D	33 ± 1*	8.2 ± 0.3*	66 ± 2.9*
	STING KO -N	46 ± 4	3.0 ± 0.1	9.4 ± 0.5
	STING KO - D	30 ± 2*	8.1 ± 0.1*	65 ± 1.0*
	$STING^{Gt}$ - N	41 ± 4	3.1 ± 0.1	9.6 ± 0.7
	$STING^{Gt}$ - D	30 ± 3*	8.2 ± 0.3*	66 ± 2.5*

Data are expressed as mean ± SD. n = 6-10 animals in each group for long term studies, n = 33-40 animals in each group for short term studies. *$p < 0.0001$ compared with appropriate N from each group, ordinary one-way ANOVA - Tukey's multiple comparisons test. Non-diabetic (N), Diabetic (D), Wildtype (WT).
